# Innate lymphoid cells are activated in HFRS, and their function can be modulated by hantavirus-induced type I interferons

**DOI:** 10.1371/journal.ppat.1012390

**Published:** 2024-07-22

**Authors:** Marina García, Anna Carrasco García, Whitney Weigel, Wanda Christ, Ronaldo Lira-Junior, Lorenz Wirth, Johanna Tauriainen, Kimia Maleki, Giulia Vanoni, Antti Vaheri, Satu Mäkelä, Jukka Mustonen, Johan Nordgren, Anna Smed-Sörensen, Tomas Strandin, Jenny Mjösberg, Jonas Klingström

**Affiliations:** 1 Center for Infectious Medicine, Department of Medicine Huddinge, Karolinska Institutet, Stockholm, Sweden; 2 Section of Oral Diagnostics and Surgery, Division of Oral Diagnostics and Rehabilitation, Department of Dental Medicine, Karolinska Institutet, Stockholm, Sweden; 3 Mechanistic & Structural Biology, Discovery Sciences, R&D, AstraZeneca, Gothenburg, Sweden; 4 Institut Curie, PSL University, Inserm, Immunity and Cancer, Paris, France; 5 Department of Virology, Faculty of Medicine, University of Helsinki, Helsinki, Finland; 6 Department of Internal Medicine, Tampere University Hospital, Tampere, Finland; 7 Faculty of Medicine and Health Technology, Tampere University, Tampere, Finland; 8 Division of Molecular Medicine and Virology, Department of Biomedical and Clinical Sciences, Linköping University, Linköping, Sweden; 9 Division of Immunology and Allergy, Department of Medicine Solna, Karolinska Institutet, Karolinska University Hospital, Stockholm, Sweden; Division of Clinical Research, UNITED STATES OF AMERICA

## Abstract

Hantaviruses cause the acute zoonotic diseases hemorrhagic fever with renal syndrome (HFRS) and hantavirus pulmonary syndrome (HPS). Infected patients show strong systemic inflammation and immune cell activation. NK cells are highly activated in HFRS, suggesting that also other innate lymphoid cells (ILCs) might be responding to infection. Here, we characterized peripheral ILC responses, and measured plasma levels of soluble factors and plasma viral load, in 17 Puumala virus (PUUV)-infected HFRS patients. This revealed an increased frequency of ILC2 in patients, in particular the ILC2 lineage-committed c-Kit^lo^ ILC2 subset. Patients’ ILCs showed an activated profile with increased proliferation and displayed altered expression of several homing markers. How ILCs are activated during viral infection is largely unknown. When analyzing PUUV-mediated activation of ILCs *in vitro* we observed that this was dependent on type I interferons, suggesting a role for type I interferons—produced in response to virus infection–in the activation of ILCs. Further, stimulation of naïve ILC2s with IFN-β affected ILC2 cytokine responses *in vitro*, causing decreased IL-5 and IL-13, and increased IL-10, CXCL10, and GM-CSF secretion. These results show that ILCs are activated in HFRS patients and suggest that the classical antiviral type I IFNs are involved in shaping ILC functions.

## Author summary

Hemorrhagic fever with renal syndrome (HFRS) and hantavirus pulmonary syndrome (HPS) are acute zoonotic diseases caused by orthohantaviruses. Hantaviruses initially infect the lung and then spread systemically, primarily infecting endothelial cells. The innate lymphoid cell (ILC) family consists of ILC1, ILC2, ILC3, and natural killer (NK) cells. While NK cells are well studied in viral infections, less is known regarding the other ILCs. Hantavirus-infected patients show highly activated immune cells, including NK cells, indicating that also other innate lymphoid cells (ILCs) might be affected. We discovered that Puumala virus (PUUV)-infected HFRS patients show increased frequency of ILC2s in circulation, and that ILCs are activated with an altered homing receptor expression pattern. Little is known regarding how ILCs are activated during viral infection. When analyzing, *in vitro*, for mechanisms behind activation we observed a PUUV-induced type I IFN-dependent activation of ILCs and that IFN-β affects ILC2 cytokine responses. These findings suggest that ILC functions are affected by classical antiviral type I IFNs.

## Introduction

Innate lymphoid cells (ILCs) play important roles in the modulation of immune and inflammatory responses [1]. Naïve ILCs (nILC) constitute an immature ILC subset [2,3] that can, at least in mice, home from peripheral blood to tissue, where they give rise to the mature ILC subsets [4]. Mature ILCs are classified in five main subsets: natural killer (NK) cells, ILC1s, ILC2s, ILC3s, and lymphoid tissue inducer (LTi) cells [5,6]. NK cells share the same features as ILC1s, but can in addition kill virus-infected cells [6]. ILC1s and ILC3s are mainly found in mucosal tissues while NK cells and ILC2s are found both in tissues and in peripheral blood [2,4]. Furthermore, two functionally distinct subsets of ILC2s can be found in peripheral blood: the more ILC2 lineage-committed c-Kit^lo^ ILC2 and the plastic c-Kit^hi^ ILC2s [7].

While NK cells have been extensively described in different viral infections [8–10], less is known regarding non-NK ILCs (hereinafter referred to as ILCs) in this context. In HIV-1 infected individuals, levels of ILCs were found to be reduced in ileum and colon [11] as well as in circulation, and to negatively correlate with viral load [12]. Total peripheral ILC levels were also found to be decreased in SARS-CoV-2-infected coronavirus disease-19 (COVID-19) patients, with ILC2s levels being decreased in severe but not in moderate disease [13–15]. Moreover, in infants with respiratory syncytial virus-caused bronchiolitis, elevated levels of ILC2s in the airways were found to associate with disease severity [16]. Overall, these studies suggest that viral infection can have an effect on the ILC landscape, but the mechanisms behind virus-induced activation of ILCs is largely unknown.

Rodent-borne hantaviruses can cause hemorrhagic fever with renal syndrome (HFRS) in Eurasia and hantavirus pulmonary syndrome (HPS) in the Americas, with case fatality rates of 0.4–10% depending on the specific HFRS-causing hantavirus, and 35%, respectively [17–20]. Humans are normally infected via inhalation of hantavirus-infected rodent excreta, and the virus then spreads systemically, primarily infecting endothelial cells [17,21]. Puumala virus (PUUV) is endemic in Europe, where it is the most common causative agent of HFRS [18,22]. HFRS patients initially develop non-specific symptoms such as high fever and headache, often progressing to kidney dysfunction and gastrointestinal symptoms, including abdominal pain, diarrhea, vomiting, and gastrointestinal bleeding. Lung dysfunction is common in HPS but can also occur in severe HFRS [19,23].

Hantaviruses are believed to trigger immunopathogenic responses that contribute to hyperinflammation [24–28]. However, the exact mechanisms leading to these events remain to be understood [22,29,30]. Several different types of immune cells have been shown to be altered in patients. Peripheral NK cells show signs of strong activation and proliferation in the acute phase of HFRS [31–33]. B and T cells are highly expanded in the circulation of HFRS and HPS patients, with concomitant elevated levels of CD8^+^ T cells in the respiratory airways [24,34–37]. Mucosa-associated invariant T (MAIT) cells were recently shown to be reduced, but highly activated, in circulation during PUUV-caused HFRS [27]. Levels of neutrophils are highly increased and also strongly activated in hantavirus-infected patients [38–40]. Mononuclear phagocytes are susceptible to hantavirus infection, and their activation and redistribution from circulation towards the airways and kidneys in HFRS patients have been reported [41–43]. Combined, these reports show that hantaviruses trigger strong immune cell responses, indicating a possible effect also on ILCs.

Type I IFNs comprise a wide array of interferons, including IFN-β and several subtypes of IFN-α. Type I IFNs have several antiviral effects; both direct, by inducing an antiviral state in exposed cells, and indirect, by further activating antiviral responses in immune cells [44,45].

Here we performed a detailed characterization of peripheral blood ILCs and NK cells, as well as of their cytokine and chemokine milieu, in PUUV-infected HFRS patients. We observed increased plasma levels of inflammatory proteins, including ILC-associated cytokines. We showed that NK cell frequencies are reduced during acute HFRS but recover during convalescence. While total ILC frequencies did not change, we report an increased frequency of ILC2s, in particular of the ILC2 lineage-committed c-Kit^lo^ ILC2 subset, and a concomitant decreased frequency of nILCs during acute HFRS. Furthermore, NK cells and ILCs displayed an activated and proliferating phenotype during acute HFRS. We observed a negative correlation between viral load and the frequencies of both NK cells and ILCs in acute HFRS, suggesting a potential direct or indirect influence of hantaviruses on the ILC landscape. Finally, *in vitro* studies showed that PUUV-mediated activation of ILC2s is dependent on type I IFNs, and that IFN-β *per se* impacts ILC2 functions by inducing specific cytokine responses.

## Material and methods

### Ethics statement

The study was approved by the Ethics Committee of Tampere University Hospital (ethical permit nr. R04180) and all subjects provided written informed consent. Control peripheral blood mononuclear cells (PBMCs) were obtained from the Blood Transfusion Clinic at the Karolinska University Hospital Huddinge, Stockholm, Sweden (ethical permit nr. 2020–02604).

### Patient samples

17 patients, 12 females and 5 males, with serologically confirmed acute HFRS were included in the study. The patients were diagnosed at the Tampere University Hospital in Finland during 2002–2007. Whole blood samples were collected, and PBMCs were isolated as previously described and stored in liquid nitrogen at -150°C, while plasma was stored at -80°C, until further use [43].

The samples were collected in the acute phase of disease (5–8 days after onset of symptoms), during the early convalescence phase (20–27 days after onset of symptoms), and at a late convalescence phase (at 180 or 360 days after onset of symptoms). Patients were stratified as having either mild or severe HFRS based on a scoring system adapted from the sequential organ failure assessment scoring system, where the maximum levels of creatinine (4 = > 440, 3 = 300–440, 2 = 171–299, 1 = 110–170, and 0 = < 110 μmol/l), minimum level of platelets (4 = < 20, 3 = 20–49, 2 = 50–99, 1 = 100–150, and 0 = > 150 x 10^3^/μl), and minimum mean arterial blood pressure (1 = < 70 and 0 = ≥ 70 mmHg) were ranked. A total score of ≥ 5 was considered severe and a score < 5 was considered non-severe HFRS [43,46].

Control PBMCs were obtained from buffy coats of 10 blood donors. PBMCs were isolated from the buffy coats by density centrifugation using Lymphoprep (StemCell Technologies), according to the manufacturer’s guidelines and stored at -150°C until further use.

### Viral load in plasma

RNA was isolated from 140 μl EDTA plasma using a column-based RNA isolation kit, according to the manufacturer’s instructions (Viral RNA mini kit, Qiagen). Isolated RNA was subjected to PUUV S RNA RT-qPCR analysis based on a previously described protocol [47], with TaqMan fast virus 1-step master mix (ThermoFisher Scientific) and using AriaMx instrumentation (Agilent).

### Flow cytometry

PBMC samples were thawed in RPMI (Cytiva) complete media [L-glutamine (ThermoFisher Scientific), 10% FCS (Sigma-Aldrich), penicillin/streptomycin (P/S) (Cytiva)] with 10 U/mL DNase (Roche) and counted. 3–4 million cells per sample were stained. Briefly, cells were incubated with LIVE/DEAD Fixable Green Dead Cell Stain Kit (ThermoFischer Scientific) and fluorochrome-conjugated antibodies directed against surface markers (S1 Table) for 20 min at room temperature in the dark followed by 2 washes with flow cytometry buffer (PBS with 2 mM EDTA). Cells were then fixed and permeabilized using FACS Lysing solution and FACS Permeabilizing solution (BD Biosciences), and next incubated with intracellular staining antibodies (S1 Table) for 30 min at 4°C in the dark. Cells were then washed and resuspended in flow cytometry buffer. Samples were acquired with a BD LSR Fortessa (BD Biosciences) flow cytometer. Flow cytometric analysis was performed using FlowJo version 10.7.2 (TreeStar, Ashland). To ensure unbiased manual gating, a blinded analysis was implemented, whereby all FCS3.0 files were renamed and coded by one person and blindly analyzed by another person. All samples were compensated electronically, and gatings were based on fluorescent-minus-one (FMO) or negative controls. After all gatings were performed, samples were decoded and data analysis was performed.

ILCs were defined as live (DCM^-^) CD1a^-^ CD14^-^ CD19^-^ CD34^-^ CD123^-^ BDCA2^-^ FcεR^-^ TCRα/β^-^ TCRγδ^-^ CD3^-^ CD45^+^ CD127^hi^ cells. NK cells were defined as DCM^-^ CD1a^-^ CD14^-^ CD19^-^ CD34^-^ CD123^-^ BDCA2^-^ FcεR^-^ TCRα/β^-^ TCRγδ^-^CD3^-^ CD45^+^ CD56^+^ CD127^lo/hi^ cells. For a detailed gating strategy see S1 Fig.

### Multiplex immunoassays

Plasma levels of IL-5, IL-6, IL-7, IL-10, IL-13, IL-15, IL-17A, IL-18, IL-23, IL- 25, IL-33, IFN-γ, TNF, CCL20, CCL27, CCL28, GM-CSF, TSLP, and granzyme A were measured in plasma diluted 1:1 using a custom made Magnetic Luminex Screening assay (R&D Systems, Minneapolis) and analysed in a Magpix instrument (Luminex), according to the manufacturer’s guidelines. Values below the lower limit of detection were replaced by half of the value of the lowest detectable value for the given soluble factor measured.

Levels of IL-4, IL-5, IL-6, IL-13, IL-15, CXCL10, and GM-CSF in ILC2 supernatants were measured as described above and analysed in a Bio-plex 200 instrument (BIO-RAD).

### ELISA

IFN-α levels in plasma and supernatants were analysed using human IFN-α pan ELISA development kit (Mabtech) according to the manufacturer’s guidelines. Plasma was diluted 1:1 in ready-to-use ELISA diluent (Mabtech) prior to the ELISA. IFN-β levels in supernatants were analysed using IFN-β DuoSet ELISA development kit (R&D Systems) according to the manufacturer’s guidelines.

### Enrichment and sorting of ILC2s

PBMCs were isolated from buffy coats from uninfected controls using Ficoll gradient density centrifugation. PBMCs were then depleted of T cells using an anti-CD3 microbead kit and LD columns and enriched for CD127-expressing cells using a CD127 microbead kit and LS columns (all Miltenyi Biotech) according to the manufacturer’s instructions. CD3-depleted and CD127-enriched PBMCs were surface stained for 30 min at 4°C. ILC2s were then sort-purified as live (DCM^-^) CD1a^-^ CD14^-^ CD19^-^ CD34^-^ CD94^-^ CD123^-^ BDCA2^-^ FcεR^-^ TCRα/β^-^ TCRγδ^-^ CD3^-^ CD45^+^ CD127^hi^ CD161^+/-^CD117^+/-^ CRTH2^+^ cells and collected in collection media [Yssel’s-supplemented IMDM (Gibco) supplemented with 1% P/S (Merck) and 2% normal human serum (NHS) (Merck)]. Cells were sorted using SONY MA900 Multi-Application Cell Sorter (SONY). See S2 Table for a full list of antibodies used.

### ILC2 *in vitro* expansion

Sorted human ILC2s were seeded at 1,000 cells per well in U-bottom 96-well plates in expansion media [collection media supplemented with 50 ng/mL of IL-1β (BioTechne) and 100 U/mL of IL-2 (Peprotech)] and incubated at 37°C. At day 5–6 half of the media was renewed with fresh expansion media. At day 10–11 cells were counted and split at a 1:3–1:4 ratio. Finally, at day 13–14 cells were counted and seeded at a concentration of 250,000 cells per well in new 96-well plates in resting medium [collection media supplemented with 2 U/mL of IL-2 (Peprotech) and 5 ng/mL of IL-7 (BioTechne)] to bring them to a resting phase. Cells were incubated for 24 h at 37°C and were then either directly used for subsequent experiments or stored at -150°C until further use.

### Cells and viruses

Primary human umbilical vein endothelial cells (HUVECs) (pooled donors, sex: mixed) (Lonza) were maintained in EGM-2 medium supplemented according to the manufacturer’s guidelines (Lonza), with the exception that cortisone was only included in the medium until cells were split to plates used for infection experiments. Cells were cultured at 37°C in 5% CO_2_. PUUV strain CG-1820 was propagated as previously described [27].

### *In vitro* co-culture of ILC2s and HUVECs

HUVECs (0.2x10^6^ cells per well in a 24-well plate) were infected with PUUV at multiplicity of infection (MOI) 1 with gentle shaking every 15 min, or left unstimulated in infection medium [HBSS (Gibco), 1% FCS (Sigma-Aldrich), 2% HEPES 1M (HyClone), 1% P/S (Cytiva)]. After 1 h of incubation at 37°C, the infection medium was removed and fresh EGM-2 medium added to the cells. After 48 h, media was renewed and 24 h later (day 3 after infection) expanded and rested ILC2s (0.1x10^6^ cells) were added to uninfected and PUUV-infected HUVECs. In parallel, cell culture supernatants from uninfected and PUUV-infected HUVECs were collected, centrifuged to remove cell debris, and added to ILC2s (0.1x10^6^ cells per well in a 24-well plate). After 24 h of culture, ILC2s from the different conditions were washed, stained (panel described in S3 Table), fixed, and acquired as described above in the flow cytometry section.

### Blocking assay

Blocking antibodies against IL-6 (Mabtech, 5 ug/mL), IL-12 (p40/p70) (Miltenyi Biotec, 5 ug/mL), IL-18 (MBL International Corporation, 5 ug/mL), mouse monoclonal isotype control IgG1κ (Biolegend, 5 ug/mL), or recombinant viral B18R protein [a decoy receptor that blocks type I IFNs (R&D systems, 1 ug/mL)] were added to the fresh supernatants collected as described above, and incubated for 4–5 h in 24-well plates at 37°C prior to experiments. ILC2s (0.1x10^6^ cells per well in a 24-well plate) were then added to the supernatants with the different blocking reagents. After 24 h of incubation, cells were washed, stained, fixed, and acquired as described in the flow cytometry section.

### IFN-β stimulation of ILC2s

Sorted and expanded frozen human ILC2s were thawed and stimulated with 10-fold dilutions (1, 10, and 100 ng/mL) of recombinant human IFN-β (Peprotech). After 24 h, ILC2s were washed and stained for flow cytometry, as previously described. A cocktail of alarmins [TSLP (50 ng/mL), IL-25 (50 ng/mL), and IL-33 (50 ng/mL)] plus IL-2 (10 U/mL) was used as a positive control for ILC2 activation.

In a separate experiment, freshly sorted and expanded ILC2s were stimulated with 10-fold dilutions (0.1, 1, 10, 100, and 1000 ng/mL) of recombinant human IFN-β (Peprotech). Supernatants were collected at day 1, 2, and 3 post-stimulations. Cytokine levels were analysed by multiplex immunoassay as described above. Media alone was used as negative control.

### *In vitro* hantavirus-infection of ILC2s

Sorted and expanded fresh human ILC2s were exposed to PUUV at MOI 0.3, 1, and 3, for 1h. The cells were then washed and fresh ILC2 resting medium was added to the cells. Medium was renewed at 5h post infection (hpi), 1 day post infection (dpi), and 3 dpi. Cells were sampled at 5 hpi and at 5 dpi, lyzed with Trizol and stored at -20°C. RNA was then extracted using direct-zol RNA MiniPrep Kit (Zymo Research) according to the manufacturer´s instructions, with RNA eluted in 30 μl nuclease-free water. The extracted RNA was subsequently reverse transcribed using high-capacity Reverse Transcriptase kit (Thermo Fischer Scientfic) according to the manufacturer´s instructions and stored at -20°C until further analysis.

### Real-time PCR

Real-time PCR was performed on a CFX96 Real-Time PCR System (Biorad) using iTaq Universal Probes Supermix (Biorad) with the following cycling conditions: initial denaturation at 95°C for 3 min, followed by 45 cycles of 95°C for 5 s and 60°C for 30 s.

The primers PUUV-F 5´-TGGACCCRGATGACGTTAAC-3´, [48] PUUV-R1mod 5´- CAGTGCTGACACTGTYTGTTGT-3´, PUUV-R2emod 5´-CAGTGCTGACACTGTTTGTT.

GT-3´ and probe PUUV-Pmod FAM-CACATTACAAGCAAG-MGB were used. PUUV-R1mod, PUUV-R2emod and PUUV-Pmod were modified from an earlier protocol [48]. Relative viral RNA change was calculated by comparing cycle threshold (ct) values at 5 dpi to 5 hpi, using 2^^ΔCt^.

### Statistical analysis

Statistical analyses were performed using GraphPad Prism software v.9.2 for MacOSX (GraphPad Software). Statistical differences between HFRS patients and controls were analyzed with Kruskal-Wallis test followed by Dunn’s multiple comparison post hoc test. Paired comparisons between HFRS in the different disease stages (acute, early convalescence, and late convalescence) were performed using Wilcoxon test. Paired t-test were performed to compare different treatments in *in vitro* assays. Spearman’s rank correlation coefficient was used for assessing correlations. Two-tailed p-values < 0.05 were considered statistically significant. Spearman’s correlation matrixes were generated with R (v.4.1.1; R Core Team, 2020) using the package corrplot (v.0.9). Principal component analysis (PCA) was performed in R (v.4.1.1) using packages Factoextra (v.1.0.7), FactoMineR (v.2.4), RColorBrewer (v.1.1–2), and ggplot2 (v.3.3.5). Data were normalised in R using the scale argument within the PCA function. Where data were missing, the values were imputed using the package missMDA (v.1.18).

### Role of the funding sources

The funders of this study had no role in the study design, data collection, data analysis, data interpretation, or writing of the report.

## Results

### Study design and patient characteristics

A total of 17 hospitalized PUUV-infected HFRS patients and 10 uninfected controls were included in the study (Table 1).

**Table 1:** Clinical and laboratory characteristics of HFRS-patients and controls.

Characteristic	HFRS patients	Controls
Number of individuals	17	10
Age in years, median (IQR)	35 (28.5–51)	47.5 (38.25–53.25)
Female, *n* (%)	12 (71)	2 (20)
Viral load at time of sampling (PUUV S RNA copies/mL), median (IQR)	94,272 (47,300–187,975)	NA
Platelet count (x 10^9^/L), median (IQR)	97 (85–119)	ND
CRP (mg/L), median (IQR)	58.2 (33.4–93.5)	ND
Creatinine (μmol/L), median (IQR)	146 (96–298)	ND
Days after symptoms onset, median (IQR)	6 (6–8)	NA
Days hospitalized, median (IQR)	5 (4–6.5)	NA
Hematocrit (L/L) median (IQR)	0.37 (0.37–0.41)	ND
PCR positivity CMV, *n* (%)	0 (0)	ND
PCR positivity EBV, *n* (%)	1 (6)	ND

NA: not applicable; ND: not determined; IQR: interquartile range; CRP: C-reactive protein; CMV: cytomegalovirus; EBV: Epstein-Barr virus.

Platelet count; normal range 150–360 x10^9^/L.

Plasma CRP; reference <3 mg/L.

Plasma creatinine; reference <90 μmol/L for women, <100 μmol/L for men.

**Fig 1:**
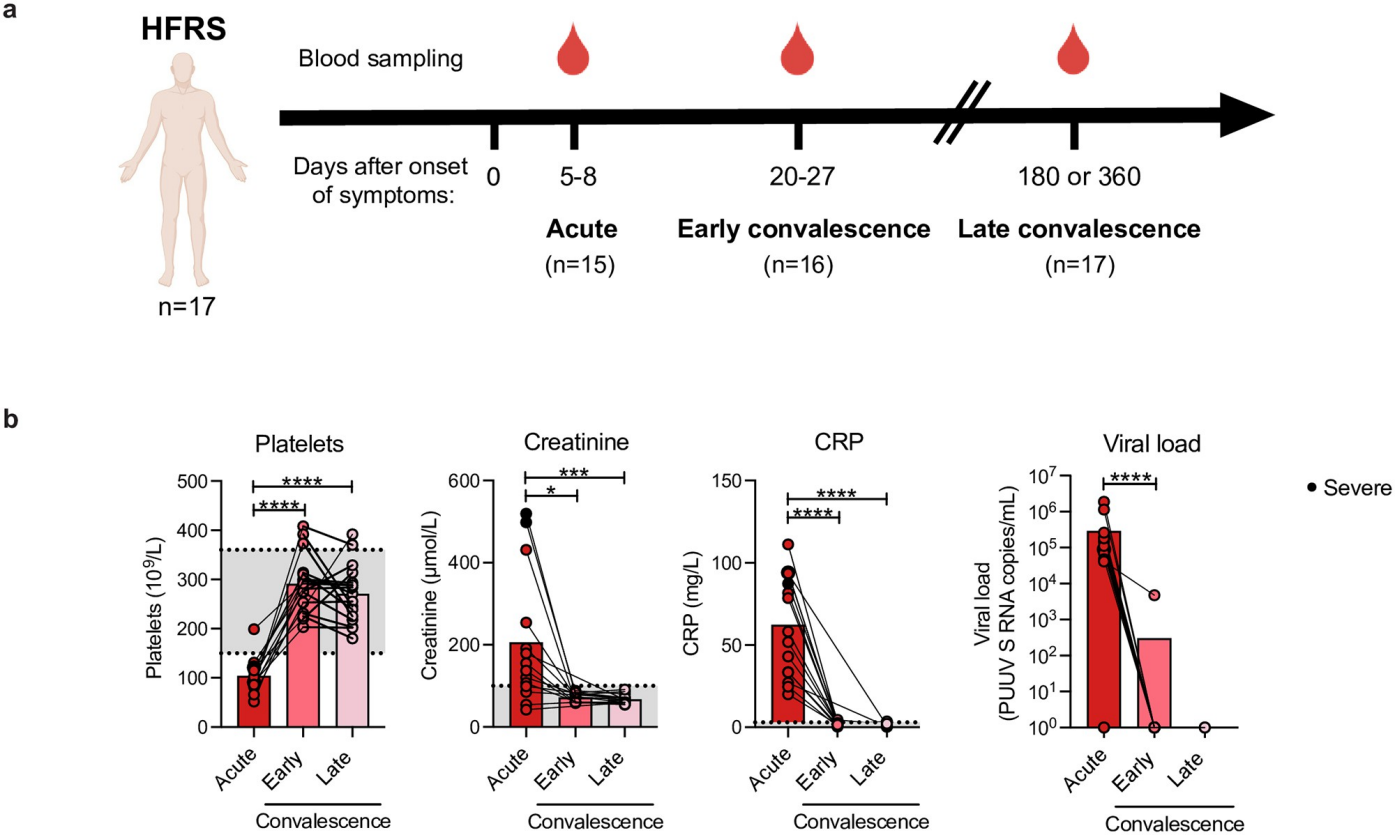
Cohort design and clinical characterization of PUUV-infected hemorrhagic fever with renal syndrome (HFRS) patients. **(a)** Schematic overview of peripheral blood sample collection in PUUV-infected HFRS patients (n = 17) at acute (5 to 8 days), early convalescence (20 to 27 days), and late convalescence phase (180 or 360 days post onset of symptoms). **(b)** Levels of platelets (10^9^/L), creatinine (μmol/L), C-reactive protein (CRP, mg/L), and copy numbers of PUUV S RNA in plasma (copies/mL) in acute (n = 15), early convalescence (n = 16), and late convalescence (n = 17) phase. Dotted lines indicate reference values in adults. Bar graphs are shown as mean and lines connect paired samples from the same patient (circles). Statistical significance was assessed using the Wilcoxon signed-rank test. Severe patients are indicated by a black circle. *p < 0.05; **p < 0.01; ***p < 0.001; ****p < 0.0001. The clip-art within the Fig 1 panel was made on BioRender.com.

Peripheral blood samples were obtained from 15 patients at the acute (5–8 days after onset of symptoms), 16 patients at the early convalescent (20–27 days after onset of symptoms), and 17 patients at the late convalescent (180 or 360 days after onset of symptoms) phase of disease (Fig 1A). Samples were not available from two patients during the acute phase and from one patient during the early convalescence phase. Hospitalized HFRS patients showed a typical clinical presentation during the acute phase, with thrombocytopenia, elevated C-reactive protein (CRP) and plasma creatinine levels, and viremia (Fig 1B, and Tables 1 and S4). For most of the patients all parameters normalized to levels within the normal range during the convalescent phase of disease (Fig 1B). The severity of the patients was assessed with a scoring system based on platelet counts, creatinine values, and mean arterial blood pressure values, as previously described [43,46]. Two patients scored as severe, while all other scored as non-severe (S4 Table).

### HFRS patients present a strong inflammatory response during the acute phase of disease

**Fig 2:**
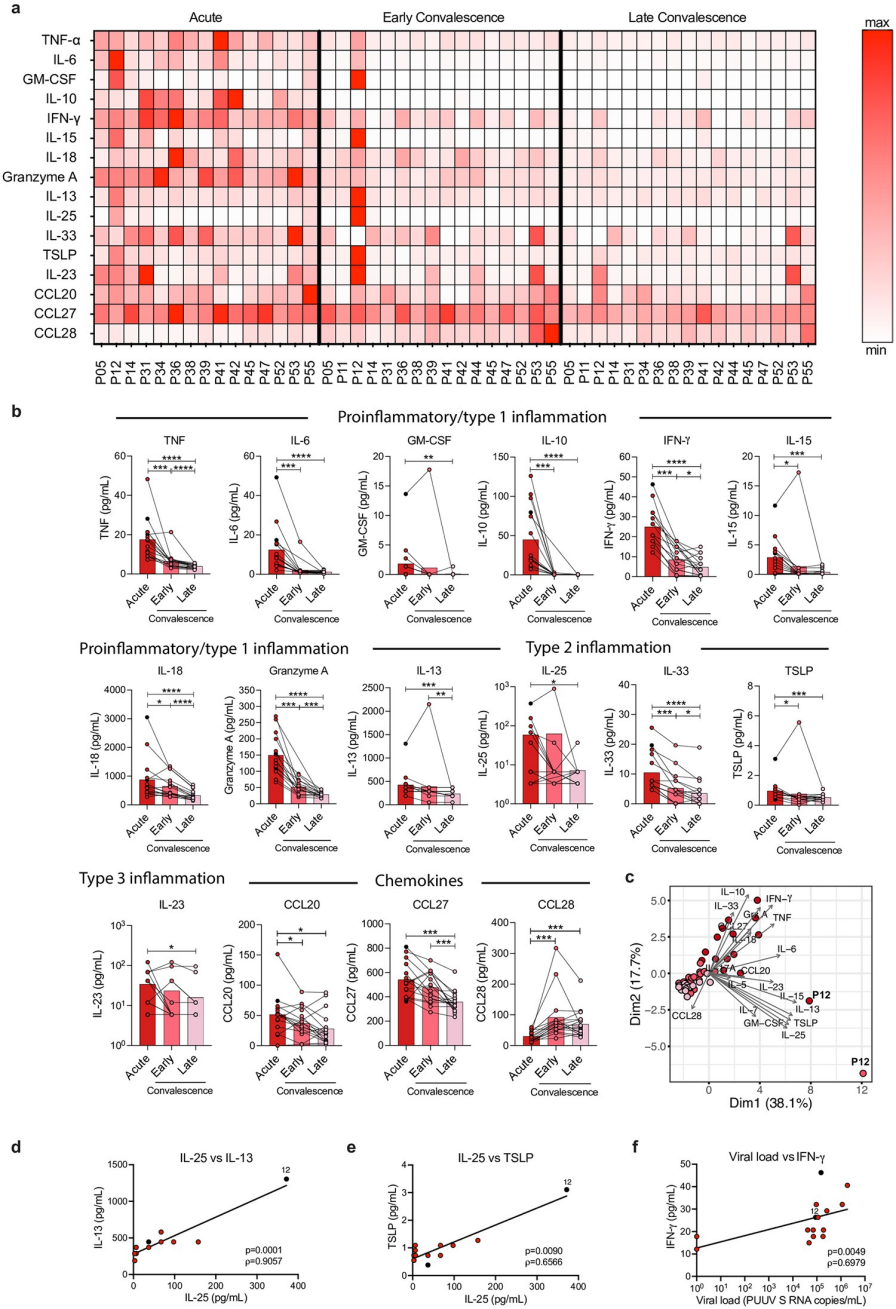
Altered levels of soluble factors in peripheral blood of HFRS patients. **(a)** Heatmap displaying the normalized plasma concentration of each soluble factor in each HFRS patient at acute, early convalescent, and late convalescent phase, as measured by multiplex immunoassay. Colors depict high (red) or low (white) concentration. Values were normalized dividing each one by the highest value obtained for the given soluble factor. **(b)** Level of soluble factors in plasma of HFRS patients in acute (n = 15), early convalescence (n = 16), and late convalescence (n = 17) phase. Bar graphs are shown as mean and lines connect paired samples from the same patient (circles). **(c)** Principal component analysis (PCA) displaying the distribution of HFRS patients according to the plasma level of soluble factors. **(d-f)** Spearman rank correlation between plasma levels of (**d)** IL-25 and IL-13, (**e**) IL-25 and TSLP, and (**f)** IFN-γ and viral load in acute HFRS patients (n = 15). Abbreviations: IFN-γ: interferon gamma; IL: interleukin; TNF: tumor necrosis factor alpha; TSLP: Thymic stromal lymphopoietin; GM-CSF: granulocyte-macrophage colony-stimulating factor; CCL: chemokine ligand. Statistical significance was assessed using the Wilcoxon signed-rank test. Severe patients are indicated by a black circle and patient 12 is labeled as P12. ρ: Spearman´s rank correlation coefficient. *p < 0.05; **p < 0.01; ***p < 0.001; ****p < 0.0001.

Hantavirus-caused disease is characterized by strong systemic inflammatory responses [25–28,49]. Using a multiplex immunoassay, we assessed the plasma levels of 19 cytokines in HFRS patients during the acute and convalescent phase of disease (Figs 2A and 2B, and S2A). As previously reported [25–28,49], the plasma levels of tumor necrosis factor (TNF), interleukin (IL)-6, granulocyte-macrophage colony-stimulating factor (GM-CSF), IL-10, interferon gamma (IFN-γ), IL-15, IL-18, and granzyme A (GrzA) were all significantly higher in acute HFRS as compared to the convalescent phases (Fig 2B). Further, we observed significantly higher levels of the type 2-associated cytokines IL-13, IL-25 (also called IL-17E), IL-33, and thymic stromal lymphopoietin (TSLP), related to ILC2 and T helper 2 cell activity and involved in functions such as tissue repair [50,51]. We also observed significantly higher levels of the type 3-associated cytokine IL-23 in the acute phase of HFRS (Fig 2B). IL-23 is involved in the activation of immune cells such as ILC3s and T helper 17 cells, which have a role in protection from tissue damage [5,52]. Moreover, we observed that levels of the chemokines CCL20 and CCL27 were significantly higher in acute samples, while the level of CCL28 was significantly decreased (Fig 2B). No differences in levels of IL-5, IL-7, and IL-17A were observed during the acute compared to later stages of HFRS (S2A Fig).

Principal component analysis (PCA) showed that samples from the acute phase separated from samples from the early and late convalescent phase. This separation was mainly driven by IL-10, GrzA, IFN-γ, TNF, IL-18, CCL27, and IL-33 (Fig 2C). Interestingly, patient 12, one of the two most severely ill patients in the cohort, deviated from the rest of the patients, both in the acute and early convalescent phase (Fig 2C). This patient showed the highest levels of several soluble factors and presented higher levels of many of them in the early convalescent phase than in the acute phase (Fig 2A), suggesting a longer than usual acute phase. We observed significant positive correlations between the type 2-associated cytokines IL-25 and IL-13 and between IL-25 and TSLP during the acute phase of HFRS (Figs 2D and 2E, and S2B).

Out of the 15 acute HFRS patients, 13 were positive for PUUV S RNA in blood. During the acute phase, a strong positive correlation was observed between viral load and plasma levels of IFN-γ (Figs 2F and S2B).

Altogether, these results showed that PUUV-infected HFRS patients display a strong inflammatory response, including elevated levels of 16 cytokines, many of which are known to be produced by or involved in the activation of ILCs and NK cells.

### Peripheral NK cells are activated but decreased in frequency during acute HFRS

Next, we characterized the ILC and NK cell compartments in PBMCs from HFRS patients. For the identification and analysis of ILCs and NK cells, we used 18-parameter flow cytometry and a modification of a well-established gating strategy (S1 Fig) [53].

**Fig 3:**
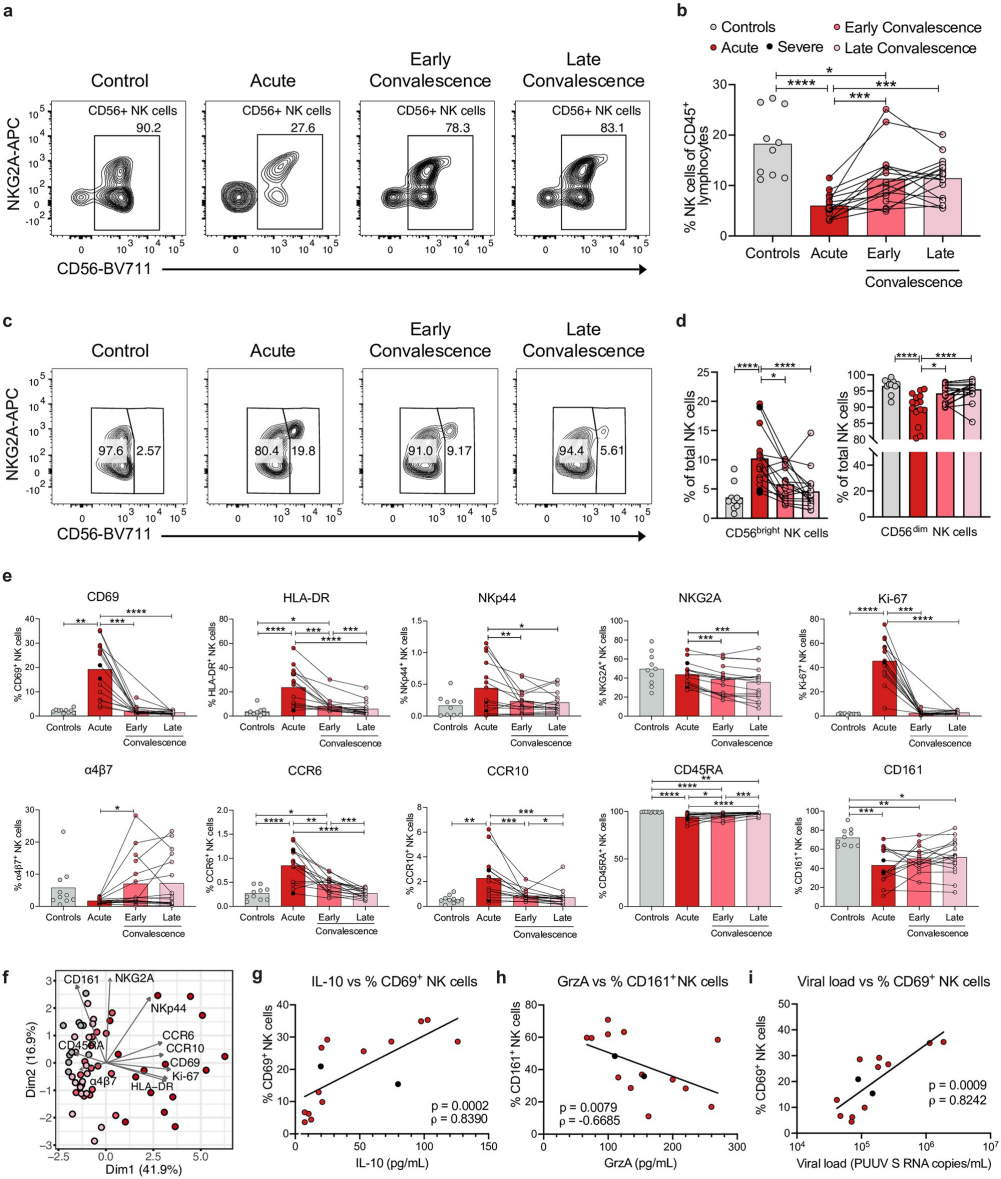
NK cells are activated, proliferating and decreased in frequency in peripheral blood of HFRS patients. **(a)** Representative flow cytometry plots showing percentage of CD56^+^ NK cells within the NKG2A^-/+^ CD127^-/+^ gate (as in S1 Fig) in a control donor and in an HFRS patient. The gates depict CD3^neg^ DCM^neg^ lineage^neg^ CD127^neg/pos^ NKG2A^neg/pos^ CD56^pos^ cells. **(b)** Percentage of NK cells out of CD45^+^ lymphocytes in control donors (n = 10) and HFRS patients during the acute (n = 15), early convalescence (n = 16), and late convalescence (n = 17) phase. **(c)** Representative flow cytometry plots showing percentage of CD56^bright^ and CD56^dim^ NK cells (gated as CD3^-^CD56^+^ cells as in S1 Fig) in a control donor and in an HFRS patient. **(d)** Percentage of CD56^bright^ and CD56^dim^ NK cells of CD3^-^CD56^+^ cells in control donors (n = 10) and HFRS patients during the acute (n = 15), early convalescence (n = 16), and late convalescence (n = 17) phase. **(e)** Percentage of CD69^+^, Ki-67^+^, HLA-DR^+^, NKp44^+^, NKG2A^+^, CCR6^+^, CCR10^+^, α4β7^+^, CD45RA^+^, and CD161^+^ NK cells of total CD3^-^CD56^+^ cells in control donors (n = 10) and HFRS patients during the acute (n = 15), early convalescence (n = 16), and late convalescence (n = 17) phase. **(f)** Principal component analysis of total CD3^-^CD56^+^ NK cells in controls and HFRS patients displaying the contribution of NK cell surface markers indicated in **(e)**. Each dot represents one donor. **(g-i)** Spearman rank correlation between **(g)** plasma IL-10 levels and the percentage of CD69^+^ NK cells, **(h)** plasma granzyme A (GrzA) levels and the percentage of CD161^+^ NK cells, and **(i)** plasma viral load (n = 13; PUUV S RNA copies/mL) and the percentage of CD69^+^ NK cells in acute HFRS patients. Bar graphs are shown as mean and lines connect paired samples from the same patient (circles). Statistical significance was assessed using the Wilcoxon signed-rank test to compare groups of HFRS patients, and the Kruskal-Wallis test followed by Dunn’s multiple comparisons test to compare controls with groups of HFRS patients. Severe patients are indicated by black circles. ρ: Spearman´s rank correlation coefficient. *p < 0.05; **p < 0.01; ***p < 0.001; ****p < 0.0001.

We observed decreased frequencies of total CD56^+^ NK cells in peripheral blood in the acute and early convalescent phase of disease, which normalized in late convalescence (Fig 3A and 3B). There was a decreased frequency of CD56^dim^ NK cells, with a concomitant increase of the smaller population of CD56^bright^ NK cells, during the acute phase of HFRS (Fig 3C and 3D). High frequencies of CD69^+^ and HLA-DR^+^ total CD56^+^ NK cells were detected in the acute phase of HFRS, indicating NK cell activation (Fig 3E). As expected from the general NK cell activation, frequencies of NKp44^+^ and NKG2A^+^ NK cells were also increased in the acute phase of HFRS (Fig 3E). Furthermore, the frequency of Ki-67^+^ NK cells was increased, suggesting that the NK cells proliferated during acute HFRS (Fig 3E). Additionally, when analyzing for homing receptors, we observed a decreased frequency of α4β7^+^ NK cells and an increased frequency of CCR6^+^ and CCR10^+^ NK cells in the acute phase of HFRS (Fig 3E), suggesting an effect on NK cell migration. The frequency of CD45RA^+^ and CD161^+^ NK cells was decreased in HFRS patients as compared to controls, with a tendency towards recovery in the convalescent phase of HFRS (Fig 3E). Analysis of surface markers in the CD56^bright^ and CD56^dim^ NK cell subsets showed similar results as observed for total NK cells (S3A and S3B Fig). PCA revealed a separation of acute HFRS from convalescent HFRS patients and from controls based on the surface markers on total NK cells (Fig 3F).

Next, we examined correlations between frequencies of NK cells and both soluble plasma proteins and clinical parameters (S4A–S4D Fig). IL-10 plasma levels positively correlated with the frequencies of activated (CD69^+^) NK cells and, although moderately, with proliferating (Ki-67^+^) NK cells (Figs 3G and S3C). GrzA levels correlated positively with the frequency of CD69^+^ NK cells (S3D Fig), and negatively with the frequency of CD161^+^ NK cells (Fig 3H). Interestingly, the frequency of activated (CD69^+^) NK cells correlated positively with viral load while the frequency of total NK cells showed a negative correlation with viral load during the acute phase of HFRS (Figs 3I and S3E).

### Peripheral non-NK cell ILCs are activated and proliferate during acute HFRS

**Fig 4:**
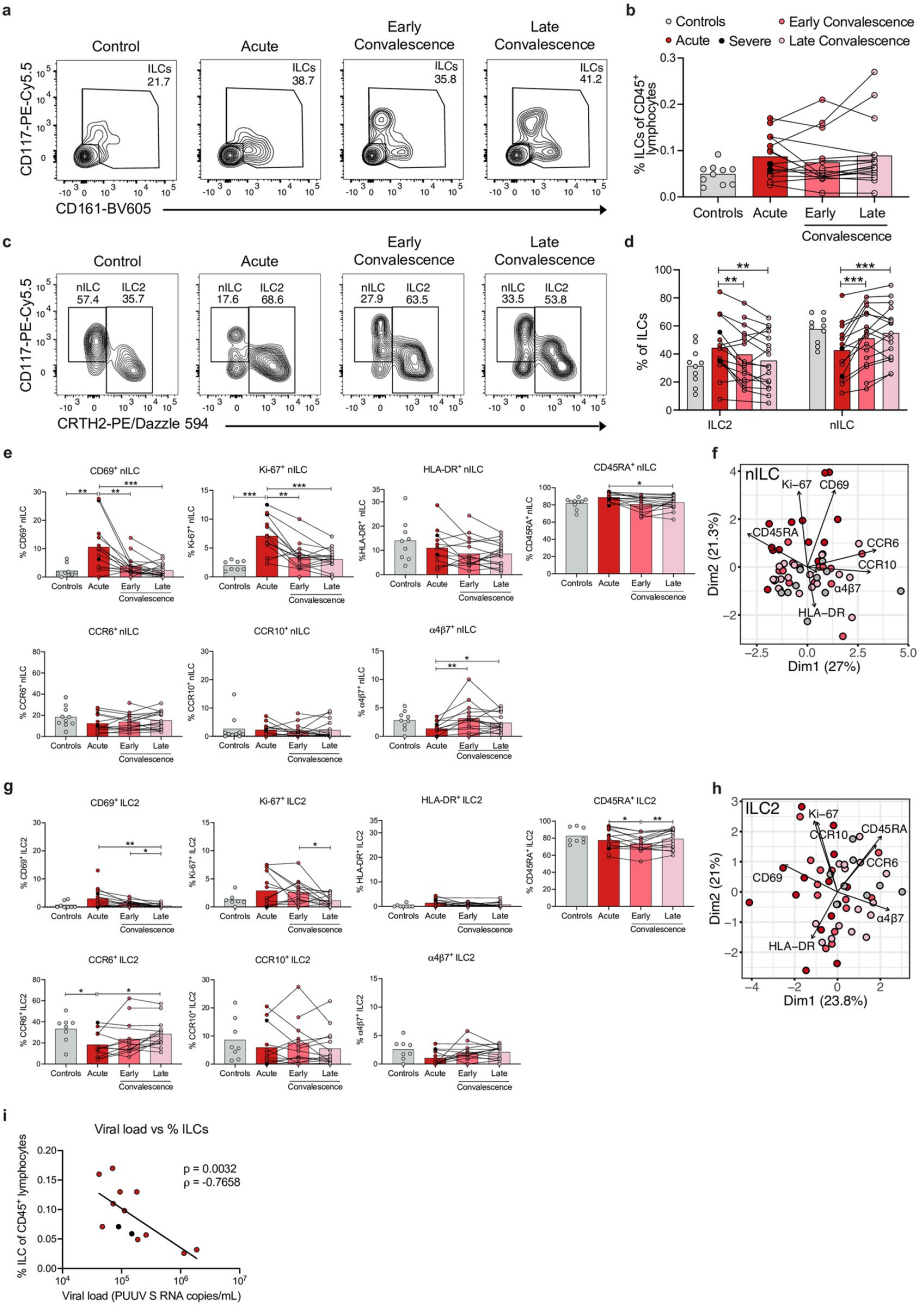
ILCs display an activated and proliferating profile in peripheral blood of HFRS patients. **(a)** Representative flow cytometry plots showing percentage of ILCs within the NKG2A^-^ CD127^hi^ gate (as in S1 Fig) in a control donor and in an HFRS patient. The gates depict CD3^neg^ DCM^neg^ lineage^neg^ NKG2A^neg^ CD127^hi^ CD161^neg/pos^ CD117^neg/pos^ cells. **(b)** Percentage of ILCs (gated as Lin^-^CD3^-^CD127^hi^ as in S1 Fig) out of CD45^+^ lymphocytes in control donors (n = 10) and HFRS patients during the acute (n = 15), early convalescence (n = 16), and late convalescent (n = 17) phase. **(c)** Representative flow cytometry plots showing percentage of ILC2 and naïve ILCs (nILC) in a control donor and in an HFRS patient (gated on ILCs as in S1 Fig). (**d)** Percentage of ILC2 and nILC in control donors (n = 10) and HFRS patients during the acute (n = 15), early convalescence (n = 16), and late convalescence (n = 17) phase. **(e)** Percentage of CD69^+^, Ki-67^+^, HLA-DR^+^, CD45RA^+^, CCR6^+^, CCR10^+^, and α4β7^+^ nILC in control donors (n = 10) and HFRS patients during the acute (n = 15), early convalescence (n = 16), and late convalescence (n = 17) phase. **(f)** Principal component analysis (PCA) of nILC in control donors and HFRS patients displaying the contribution of nILC surface markers indicated in **(e)**. **(g)** Percentage of CD69^+^, Ki-67^+^, HLA-DR^+^, CD45RA^+^, CCR6^+^, CCR10^+^, and α4β7^+^ ILC2 in control donors (n = 10) and HFRS patients during the acute (n = 15), early convalescence (n = 16), and late convalescence (n = 17) phase. **(h)** PCA of ILC2 in control donors and HFRS patients displaying the contribution of ILC2 surface markers indicated in **(g)**. **(i)** Spearman rank correlation between plasma viral load (PUUV S RNA copies/mL) and the percentage of ILCs out of CD45^+^ lymphocytes in acute HFRS patients (n = 13). Bar graphs are shown as mean and lines connect paired samples from the same patient (circles). Statistical significance was assessed using the Wilcoxon signed-rank test to compare groups of HFRS patients, and the Kruskal-Wallis test followed by Dunn’s multiple comparisons test to compare controls with groups of HFRS patients. Severe patients are indicated by a black circle. Patients with low cell numbers (fewer than 20 events) in the corresponding gate were removed from the analysis. ρ: Spearman´s rank correlation coefficient. *p < 0.05; **p < 0.01; ***p < 0.001; ****p < 0.0001.

We next characterized the peripheral non-NK cell ILC responses in the HFRS patients. No significant difference in total ILC frequency was observed between the patients and the controls (Fig 4A and 4B). Interestingly, as for NK cells, we observed a strong negative correlation between viral load and the frequency of ILCs in acute HFRS, showing reduced frequencies of peripheral ILCs in patients with higher viral loads (Fig 4I). Furthermore, we observed increased frequencies of activated (CD69^+^) and proliferating (Ki-67^+^) ILCs during the acute phase of HFRS (S5A–S5B Fig). No differences were observed in the frequencies of HLA-DR, and CD45RA-expressing ILCs (S5C–S5D Fig). When assessing expression of homing markers, we found a decreased frequency of α4β7^+^ ILCs in acute HFRS, but no significant difference in frequencies of ILCs expressing the chemokine receptors CCR6 or CCR10 (S5E–S5G Fig).

Next, we explored specific ILC subsets. CD117^neg^ ILCs in peripheral blood constitute a heterogenous population with yet undefined functions [2]. We therefore decided to focus our analysis on the more well-defined nILC and ILC2 subsets. The composition of these ILC subsets changed over time in the HFRS patients (Fig 4C and 4D). We observed an increase in ILC2 frequency and a decreased frequency of nILC in the acute phase of HFRS (Fig 4C and 4D).

### Peripheral c-Kit^lo^ ILC2s are increased in frequency during HFRS

Next, we characterized more in detail the phenotype of these ILC subsets. Increased frequencies of activated (CD69^+^) and proliferating (Ki-67^+^) nILCs were observed in acute HFRS (Fig 4E). Similar to what we observed for NK cells (Fig 3E), a decreased frequency of α4β7^+^ nILC was also observed in acute HFRS (Fig 4E). No differences were observed in the frequencies of nILCs expressing HLADR, CCR6, and CCR10 in HFRS patients as compared to controls (Fig 4E). PCA based on the frequency of expression of surface markers in nILCs showed a separation between the acute HFRS patients and controls (Fig 4F).

The ILC2 population showed a similar phenotypic pattern as the nILCs, with increased frequency of activated (CD69^+^) and proliferating (Ki-67^+^) cells during acute HFRS (Fig 4G). Additionally, a decreased frequency of CCR6^+^ ILC2s was observed in acute HFRS compared to controls (Fig 4G). No such differences were observed in the frequencies of ILC2s expressing HLA-DR, CCR10, and α4β7 (Fig 4G). In line with these findings, PCA, based on the frequency of expression of surface markers on ILC2s, showed no clear separation between the acute and convalescent HFRS. However, a separation between acute HFRS and control samples was observed (Fig 4H). When analyzing for possible correlations to soluble proteins in acute HFRS (S6A–S6C Fig), we observed a low positive correlation between the plasma levels of IL-10 and the frequency of CD69^+^ ILC2s (S5K Fig). A moderate positive correlation was also observed between plasma levels of the CCR10 ligand CCL27 and CCR10^+^ ILC2s (S5L Fig). Further, a moderate negative correlation was seen between plasma levels of TSLP and frequency of CCR10^+^ ILC2s in acute HFRS (S5M Fig). The two first-mentioned correlations were also observed for total ILCs, as well as a positive correlation between plasma levels of IL7 and the frequency of Ki-67^+^ ILCs (S5H–S5J Fig).

**Fig 5:**
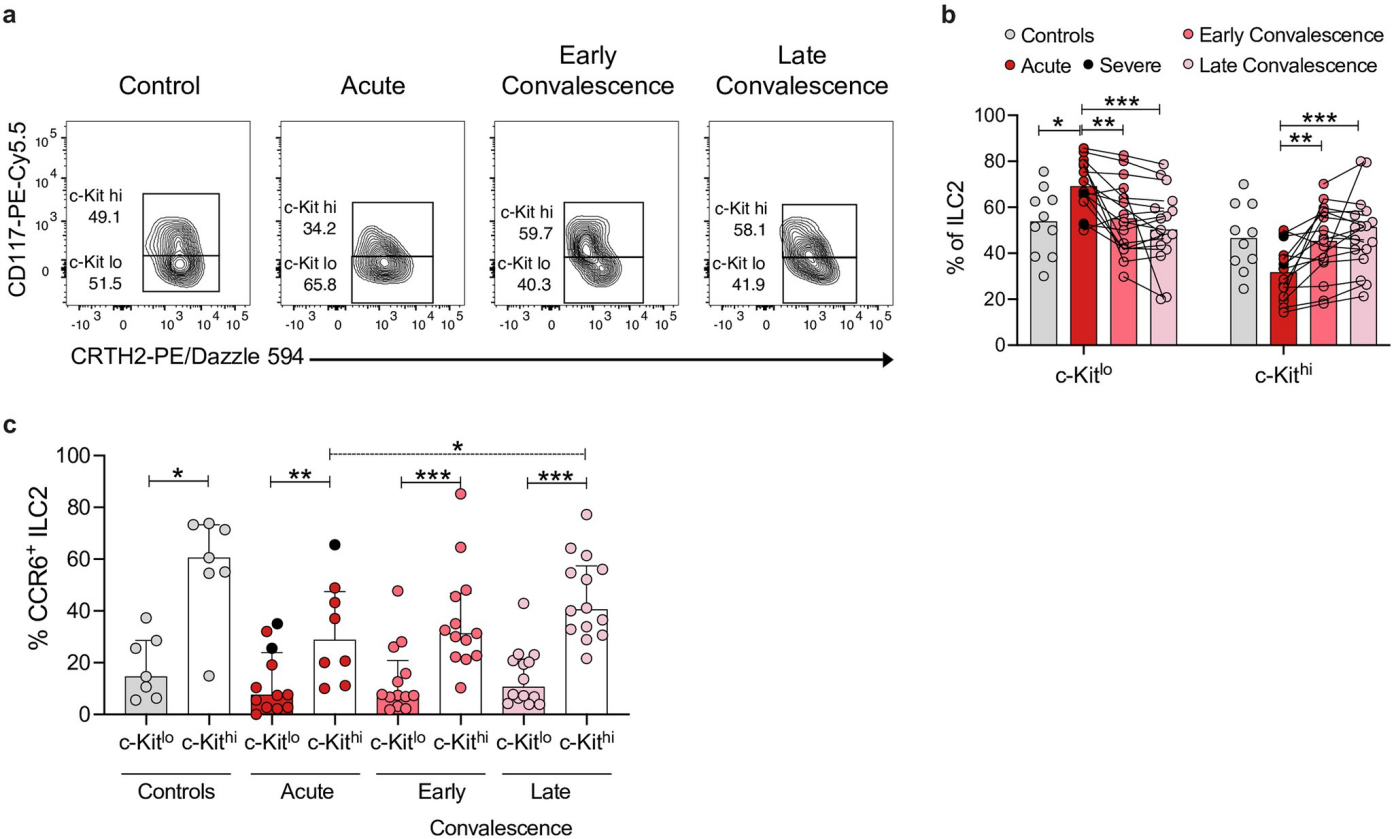
Increased ILC2 c-Kit^lo^ frequency in peripheral blood of acute HFRS patients. **(a)** Representative flow cytometry plots showing percentages of c-Kit^lo^ and c-Kit^hi^ ILC2s in a control donor and in an HFRS patient (gated on total ILC2s as in S1 Fig). **(b)** Percentage of c-Kit^lo^ and c-Kit^hi^ ILC2s in control donors (n = 10) and HFRS patients during the acute (n = 15), early convalescence (n = 16), and late convalescence (n = 17) phase. **(c)** Percentage of CCR6^+^ c-Kit^lo^ and CCR6^+^ c-Kit^hi^ ILC2s in control donors (n = 10) and HFRS patients during the acute (n = 15), early convalescence (n = 16), and late convalescence (n = 17) phase. Bar graphs are shown as mean and lines connect paired samples from the same patient (circles). Statistical significance was assessed using the Wilcoxon signed-rank test to compare groups of HFRS patients, and the Kruskal-Wallis test followed by Dunn’s multiple comparisons test to compare controls with groups of HFRS patients. Severe patients are indicated by a black circle. Patients with low cell numbers (fewer than 20 events) in the corresponding gate were removed from the analysis. *p < 0.05; **p < 0.01; ***p < 0.001.

Having observed an increased frequency of ILC2s (Fig 4D), a decreased frequency of CCR6^+^ ILC2s (Fig 4G), and increased plasma levels of type 2 cytokines in acute HFRS (Fig 2), we next assessed whether there were changes in the ILC2 subsets in HFRS patients. Indeed, we observed increased frequencies of c-Kit^lo^ ILC2s and concomitantly decreased frequencies of cKit^hi^ ILC2s during the acute phase of HFRS, as compared to convalescent HFRS patients and controls (Fig 5A and 5B). Moreover, c-Kit^hi^ ILC2s showed higher frequency of CCR6 expression than c-Kit^lo^ ILC2s, both in patients and controls (Fig 5C). And, as expected from the observed reduction of CCR6^+^ ILC2s in acute HFRS (Fig 4G), we also observed a lower frequency of CCR6^+^ c-Kit^hi^ ILC2s in the acute phase of HFRS as compared to the late convalescent phase (Fig 5C).

### ILC2s are activated by soluble factors produced by PUUV-infected endothelial cells

**Fig 6:**
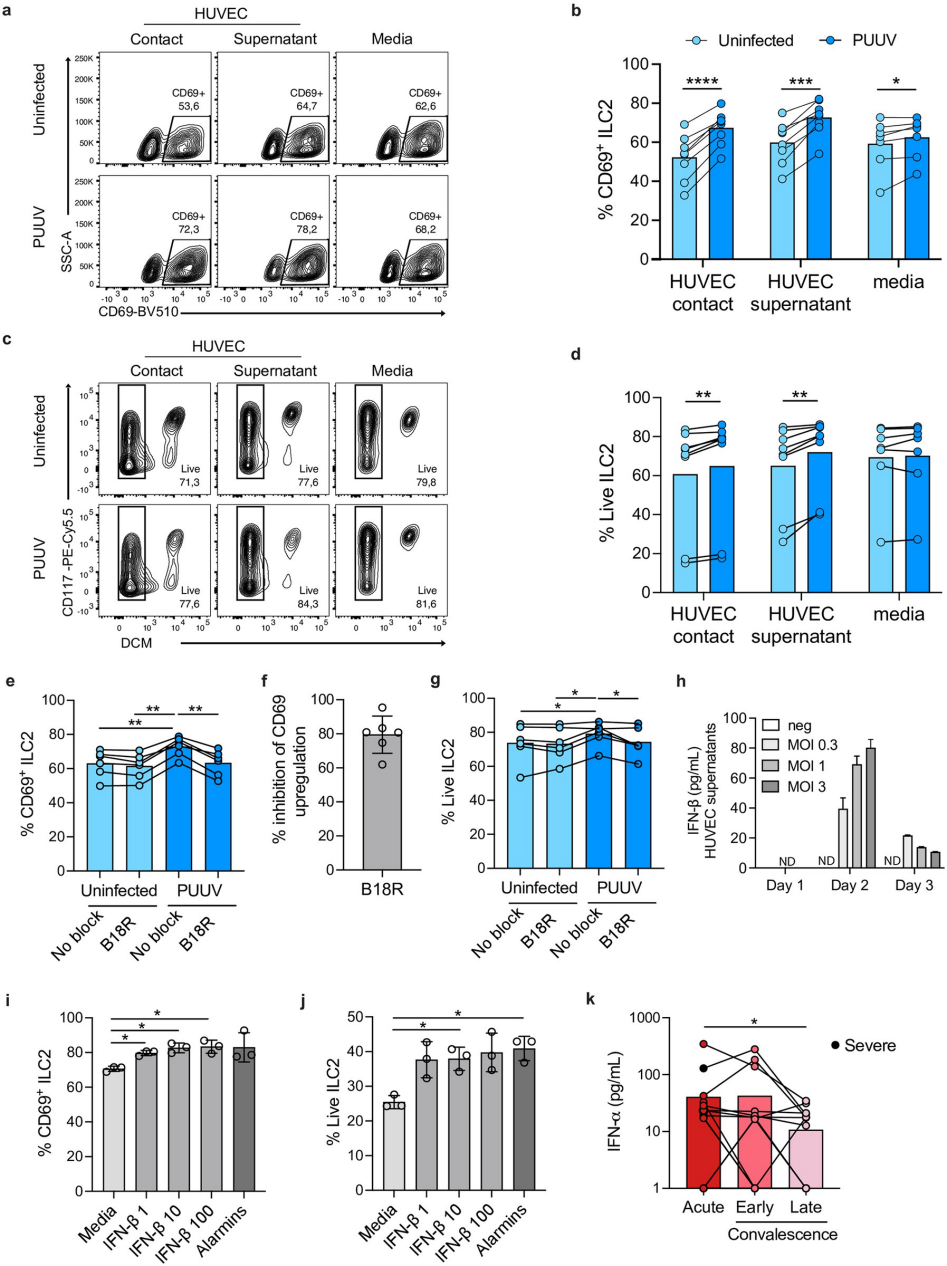
Increased activation of ILC2s co-cultured with PUUV-infected endothelial cells. **(a-b)** Representative flow cytometry plots **(a)** and graphs **(b)** showing the percentage of activation (CD69^+^) of expanded human ILC2s when co-cultured for 24 h in direct contact with uninfected or PUUV-infected HUVEC endothelial cells, with their supernatants, or exposed to PUUV or media alone (n = 8, 4 independent experiments). **(c-d)** Representative flow cytometry plots **(c)** and graphs **(d)** showing the percentage of live expanded human ILC2s when co-cultured for 24 h in direct contact with uninfected or PUUV-infected HUVEC endothelial cells, with their supernatants, or exposed to PUUV or media alone (n = 8, 4 independent experiments). **(e-g)** Type I IFN blocking reagent B18R was added to supernatants of HUVEC cultures (PUUV-infected and uninfected) for 5 h prior to addition to human expanded ILC2s (n = 6, same donors as in (a-d), 3 independent experiments) and incubation for 24 h. **(e, g)** Percentage of **(e)** activation (CD69^+^) and of **(g)** live human expanded ILC2s. **(f)** Percentage of inhibition of the increase in activation (CD69^+^) of ILC2s caused by PUUV-infected HUVEC, when pre-blocking supernatants with B18R. **(h)** Levels of IFN-β in supernatants from PUUV-infected and uninfected HUVECs over time (n = 3). ND: not detected. **(i, j)** Percentage of **(i)** activation (CD69^+^) and of **(j)** live human expanded ILC2s cultured for 24 h with increasing concentrations of recombinant human IFN-β (ng/mL), the canonical ILC2 activators (alarmins TSLP, IL-25, and IL-33) plus IL-2, or only media (n = 3, 2 independent experiments). **(k)** Levels of IFN-α in plasma of patients with HFRS during the acute (n = 15), early convalescence (n = 16), and late (n = 17) convalescent phase of disease. Black dots represent the severe HFRS patients. Bar graphs are shown as mean and lines connect paired samples from the same patient (circles). Statistical significance was assessed using the Wilcoxon signed-rank test to compare groups of HFRS patients (k), and paired t-test to compare conditions from *in vitro* assays. *p < 0.05; **p < 0.01; ***p < 0.001; ****p < 0.0001.

To investigate how ILCs are activated in the context of hantavirus infection, we performed an *in vitro* co-culture assay. Given that ILCs are scarce in peripheral blood, we enriched ILCs from buffy coats, sorted ILC2s and expanded them *ex vivo*. ILC2s co-cultured with PUUV-infected HUVECs showed an increased frequency of CD69 expression, indicating activation (Fig 6A and 6B). To test if activation was cell contact-dependent, we next exposed ILCs to conditioned medium from uninfected and PUUV-infected HUVECs. This revealed a cell contact-independent activation of ILC2s, mediated by conditioned medium from PUUV-infected HUVECs (Fig 6A and 6B). ILC2 exposed to supernatants from PUUV-infected HUVEC also showed increased viability (Fig 6C and 6D). Taken together, these results suggest that soluble factor(s) secreted by hantavirus-infected HUVECs can activate ILC2s.

### PUUV-mediated ILC2 activation is dependent on type I IFNs

To identify potential factors involved in the PUUV-mediated activation of ILC2s, we pre-treated supernatants from infected and uninfected HUVECs with cytokine-blocking compounds before supernatants were added to the ILC2s. Blocking type I IFNs significantly decreased the activation and viability of ILC2s exposed to supernatants from PUUV-infected HUVECs (Fig 6E–6G). In contrast, blocking IL-6, IL-12, and IL-18 did not affect the activation of ILC2s (S7 Fig). Endothelial cells normally respond to viral infections by producing IFN-β. [54,55] To verify that PUUV induced an IFN-β response in infected HUVECs, supernatants were collected daily for three days and subsequently analysed for IFN-β levels. IFN-β was detected in supernatants from PUUV-infected cells at days two and three post infection (Fig 6H), showing that the conditioned supernatants contained type I IFNs.

**Fig 7:**
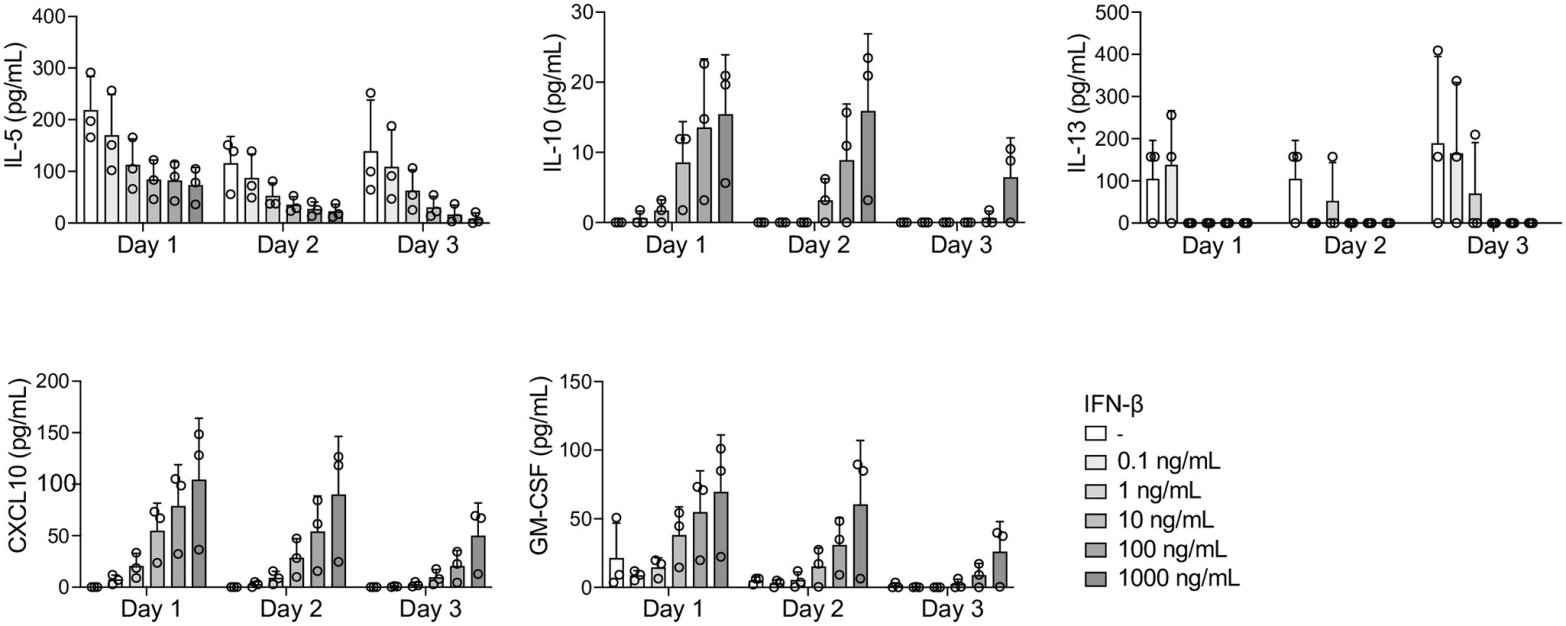
IFN-β influences ILC2 cytokine responses *in vitro*. Levels of IL-5, IL-10, IL-13, CXCL10, and GM-CSF in supernatants from ILC2s exposed to recombinant human IFN-β *in vitro*. Human ILC2s were expanded *in vitro* and exposed to IFN-β (0.1, 1, 10, 100, and 1000 ng/mL) for 3 days. Untreated cells were used as a negative control. Bar graphs are shown as mean with standard deviation (n = 3).

To verify that type I IFNs *per se* could activate ILC2s, we next exposed ILC2s to increasing concentrations of IFN-β for 24 h and then analyzed their activation status. This revealed a dose-dependent effect on the activation of ILC2s (Fig 6I) and a dose-independent increase in viability (Fig 6J). The highest concentration of IFN-β tested caused similar levels of ILC2 activation as the canonical ILC2 activators (a combination of the alarmins TSLP, IL-25, and IL-33) (Fig 6I). We next assessed IFN-β-mediated effects on ILC2 cytokine responses. To this end, supernatants from ILC2s exposed to different concentrations of IFN-β were collected daily for three days and then cytokine levels were analysed. While IL-4, IL-6, and IL-15 were not detected in supernatants from untreated or IFN-β-treated cells, supernatants from IFN-β-treated ILC2s contained elevated levels of CXCL10, IL-10, and GM-CSF, and lower levels of IL-5 and IL-13 as compared to controls (Fig 7).

We observed a small effect on activation (Fig 6B) but not on viability (Fig 6D) of PUUV-exposed ILC2s, indicating a possible direct effect of PUUV on activation. Endothelial cells are the main target for hantaviruses, but also other types of cells, including monocytes, can be infected [42]. To test if also ILC2s can be infected by hantavirus we exposed ILC2s to PUUV for 5 days and then analysed levels of PUUV RNA in the cells. Five days after infection, a 10-fold increase in PUUV RNA was detected in ILC2s from one of three donors, suggesting ILC2s may be a target for hantavirus infection (S8 Fig).

Finally, we assessed the levels of type I IFNs in HFRS patients. HFRS patients in the acute phase presented with higher levels of IFN-α in plasma as compared to late convalescent patients (Fig 6K), confirming that the level of type I IFNs is increased in HFRS.

## Discussion

Whereas NK cells have been extensively described in several human viral infections [8–10] including hantavirus infections [31–33,56–58], ILCs remain understudied in human viral infections. Recent studies reveal dysregulated frequencies and phenotypes of ILCs in HIV-1 [11,12], SARS-CoV-2 [13–15], rhinovirus [59], and respiratory syncytial virus (RSV) infection [16]. Here we provide a detailed characterization of total ILCs, including NK cells and non-NK ILCs, in PUUV-infected HFRS patients. We show that total ILCs are activated during acute HFRS and that their levels negatively correlate to the viral load. We also show *in vitro* that PUUV-infection of endothelial cells can activate ILC2s in a type I IFN-dependent manner, that PUUV can infect human ILC2s, and that IFN-β affects ILC2 cytokine responses.

HFRS is characterized by a strong general immune activation, including hyperinflammation [18,60]. Viral load normally peaks between the first 3 to 5 days of disease, and hantaviruses are normally not detected in the convalescent phase [19,22,61,62]. In line with this, the HFRS patients in our study presented with typical laboratory findings together with a strong inflammatory response in the acute phase of disease [25–28,49]. From an ILC-perspective, we observed increased levels of cytokines such as IL-13, IL-25, IL-33, and TSLP in acute PUUV-infected HFRS patients. The alarmins IL-25, IL-33, and TSLP are known activators of ILC2s, which, upon activation, can secrete IL-13 [63,64]. These alarmins are upregulated upon infection or damage of epithelial, endothelial, and stromal cells [65–67]. Recently, IL-33 was reported to be elevated in plasma of Hantaan virus-infected HFRS patients and to positively correlate with disease severity, indicating involvement in pathogenesis [68]. There are conflicting data regarding IL-5 in HFRS. While we did not observe an increase in IL-5, Shakirova and coworkers have reported elevated IL-5 levels in HFRS patients [69].

As recently shown by others [32], we observed a transient reduction in NK cell frequencies in peripheral blood of PUUV-infected HFRS patients. Moreover, as earlier shown [31], we observed that remaining circulatory NK cells were highly proliferating, with approximately half of them expressing Ki-67. During the acute phase of HFRS, NK cells also showed increased expression of several activation markers and altered expression of chemokine receptors such as CCR6 and α4β7, which are associated with migration to tissues (lung and intestines, respectively) [70–72]. Hence, NK cell migration may explain the observed decreased frequencies of NK cells in peripheral blood during the acute phase of HFRS. Interestingly, in peripheral blood viral load correlated negatively with the frequency of NK cells but positively with the frequency of activated NK cells. This suggests that viral replication indirectly impacts the activation of NK cells during HFRS. In line with this, we have previously shown that hantaviruses have strong anti-apoptotic properties that potentially protect infected cells from cytotoxic lymphocyte-mediated killing and may lead to NK cell-mediated bystander killing of uninfected cells [57,58,73,74]. We observed a decreased frequency of CD161^+^ NK cells in acute HFRS, mirroring our previous observation of downregulated CD161 expression on MAIT cells in HFRS [27].

Levels of IFN-γ positively correlated with viral load in HFRS patients. IFN-γ is a cytokine with strong antiviral effects produced by a wide array of innate and adaptive lymphocytes, including ILCs, NK cells, NKT cells, and T cells [44,75–77]. The main target of hantavirus infection, endothelial cells, and the potential target monocytes [18–42], do not express IFN-γ. It can therefore be speculated that active viral replication in infected endothelial cells indirectly, for example via enhanced IL-15 production [58], activates the IFN-γ production by NK cells and other immune cells.

The frequency of ILCs has been shown to be reduced in the circulation of individuals with acute HIV infection, and to correlate negatively with the viral load [12]. We recently reported a decrease in peripheral ILC frequencies and numbers in COVID-19 [13]. In contrast, here, we did not observe a significant change in the frequency of ILCs in HFRS. Interestingly though, and in line with that observed in HIV-infected individuals [12], frequencies of peripheral ILCs in acute HFRS showed a strong negative correlation with viral load. Furthermore, as described for moderately ill COVID-19 patients [13], acute HFRS patients presented increased frequencies of ILC2s with a concomitant reduction of nILC frequencies. In contrast to our findings in COVID-19 patients [13], we observed increased frequencies of Ki-67-expressing ILC2s and nILCs in the acute phase of HFRS, showing increased proliferation. Moreover, the decreased levels of α4β7^+^ nILCs in acute HFRS suggest that the reduced frequency of peripheral blood nILCs could be due to their migration to tissues [4]. Alternatively, nILCs might differentiate into mature ILC subsets, such as ILC2s, in circulation. However, it remains unclear if nILCs are recruited to tissues and if mature ILC subsets can be replenished by circulating nILCs in humans [4,78,79]. Acute HFRS patients presented increased frequencies of c-Kit^lo^ ILC2s. Two ILC2 subsets have been defined, differing in their surface expression of cKit and their functionality. c-Kit^lo^ ILC2s are more mature and ILC2-lineage-committed, while c-Kit^hi^ ILC2s show plasticity towards an ILC3 phenotype and functionality [7]. Moreover, c-Kit^lo^ ILC2s express lower levels of CCR6 compared to c-Kit^hi^ ILC2s [7]. In line with this, acute HFRS patients showed decreased frequency of CCR6^+^ c-Kit^hi^ ILC2s as compared to convalescent patients and controls. This suggests a skewing of c-Kit^hi^ ILC2s towards more ILC2-commited cells in HFRS, possibly explaining the increase in c-Kit^lo^ ILC2 frequencies in acute HFRS. Alternatively, this decrease in CCR6^+^ c-Kit^hi^ ILC2 levels could also be due to migration of these cells to lung, where ILC2s have been shown to play an important role in tissue repair during influenza infection in mice [64,80].

Several immune cell types have been described to be activated in HFRS and HPS patients [24,27,31,32,34,40,42]. Nonetheless, the mechanisms of their activation remain largely elusive. We recently showed that hantavirus-mediated activation of MAIT cells is type I IFN-dependent [27]. Here, we show that hantavirus can also activate ILCs via induction of type I IFNs produced by infected cells. Besides from type I IFNs, levels of a wide range of cytokines are elevated during HFRS, which likely contribute further to activation of ILCs and MAIT cells in patients. We observed that ILC2s may be infected with PUUV, suggesting ILCs are potential targets for hantavirus. Interestingly, Dengue virus, another zoonotic RNA virus, has been shown to infect ILC2s, with a majority of all circulating ILC2s being infected in some patients [81]. It remains to be investigated if ILCs are infected also in HFRS patients and what potential effects this could have on the ILC phenotype and functions.

Type I IFNs are crucial for a successful immune defense against viruses. Besides from inducing an antiviral state that interferes with viral replication, type I IFNs also stimulate immune cells, like B, T, and NK cells, to take part in the antiviral defense [45,82]. The effects of type I IFNs on immune cells are complex and context dependent; both the quantity and the timing of the IFN production impact their response, as well as other specific immune responses simultaneously taking place, for instance inflammatory responses [45]. We observed that IFN-β had a clear effect on ILC2 cytokine responses by decreasing IL-5 and IL-13 and increasing IL-10, GM-CSF, and CXCL10 secretion. IFN-β has previously been shown to decrease human and mouse ILC2 production of IL-5 and IL-13 [83,84]. Further, it has been reported that type I IFNs inhibit ILC2 proliferation in mice [83], while others—in line with our observation of increased human ILC2 survival—reported decreased apoptosis in IFN-β stimulated mouse ILC2s [84]. Of particular interest in the context of viral infections was the observation of increased IL-10 secretion by ILC2s upon IFN-β stimulation. Human ILC2s have been previously shown to acquire a regulatory phenotype when induced with retinoic acid, inhibiting the secretion of IL-5 and IL-13 while stimulating the secretion of IL-10 [85]. Further, allergic patients treated with allergen immunotherapy showed increased levels of IL-10^+^ ILC2 which, *in vitro*, attenuated type 2 T helper responses and maintained epithelial cell integrity [86]. Whether hantavirus infection could trigger a similar protective IFN-induced IL-10^+^ ILC2 phenotype in patients remains to be further investigated. In summary, the overall effect of type I IFNs on ILC2s will depend on the combined effect of the cytokine milieu induced by the infection. It remains to be studied if different viruses, which induce type I IFNs with different magnitudes and kinetics and different additional inflammatory cytokine responses, have different potential to activate or suppress ILC functions in patients.

Here we characterized peripheral ILCs in PUUV-infected HFRS patients. Future studies of ILCs in tissue samples, such as lung and intestines, can add important knowledge regarding possible ILC tissue infiltration and local ILC responses.

In conclusion, this study provides the first comprehensive characterization of total circulating ILCs in hantavirus-infected patients. We report an overall activated and proliferating ILC profile in these patients, with a particular increased frequency of the ILC2 subset, and a skewing towards the ILC2 lineage-committed c-Kit^lo^ ILC2 in acute HRFS. Further, we show that, *in vitro*, ILC2 activation and functionality can be mediated by type I IFNs secreted by PUUV-infected endothelial cells, and that ILC2 might be a potential target for hantavirus infection. Additionally, we observe that NK cells are reduced in frequencies and confirm that remaining circulating NK cells are highly activated and proliferating in acute HFRS. Moreover, we report a negative correlation between viral load and the frequencies of both NK cells and ILCs in acute HFRS, suggesting a potential influence of viral replication on these cells during the acute phase of hantavirus-caused disease.

## Supporting information

S1 Fig Gating strategy for ILC and NK cell identification in flow cytometry. Gating strategy used for the identification of ILCs and NK cells by flow cytometry.
(PDF)

S2 Fig Correlations of clinical parameters and soluble factors in plasma of HFRS patients. **(a)** Level of soluble factors in plasma of HFRS patients in acute (n = 15), early convalescence (n = 16), and late convalescence (n = 17) phase measured by multiplex immunoassay. Abbreviations: IL: interleukin. Bar graphs are shown as mean and lines connect paired samples from the same patient (circles). Statistical significance was assessed using the Wilcoxon signed-rank test. Severe patients are indicated by a black circle. **(b)** Spearman correlation matrix of the clinical parameters and the soluble markers measured in plasma of acute HFRS patients by a multiplex immunoassay. The colour of the circles indicates positive (red) and negative (blue) correlations that were statistically significant (p < 0.05) as measured by the Spearman’s rank correlation coefficient test. The colour intensity and the size of the circle are proportional to the correlation coefficients.
(PDF)

S3 Fig NK cell subsets are activated, proliferating, and present a migratory profile in peripheral blood of HFRS patients. **(a-b)** Percentage of CD69+, Ki-67+, HLA-DR+, NKp44+, NKG2A+, CCR6+, CCR10+, α4β7+, CD45RA+, and CD161+ **(a)** CD56dim NK cells and **(b)** CD56bright NK cells in control donors (n = 10) and HFRS patients during the acute (n = 15), early convalescence (n = 16), and late convalescence (n = 17) phase. **(c-e)** Spearman rank correlation between **(c)** plasma IL-10 levels and the percentage of Ki-67+ NK cells, **(d)** plasma granzyme A (GrzA) levels and the percentage of CD69+ NK cells, and **(e)** plasma viral load (n = 13; PUUV S RNA copies/mL) and the percentage of NK cells out of CD45+ lymphocytes in acute HFRS patients. Bar graphs are shown as mean and lines connect paired samples from the same patient. Statistical significance was assessed using the Wilcoxon signed-rank test to compare groups of HFRS patients, and the Kruskal-Wallis test followed by Dunn’s multiple comparisons test to compare controls with groups of HFRS patients. Severe patients are indicated by a black circle. ρ: Spearman´s rank correlation coefficient. *p < 0.05; **p < 0.01; ***p < 0.001; ****p < 0.0001.
(PDF)

S4 Fig Correlations of soluble factors and clinical parameters with NK cells in HFRS patients. Spearman correlation matrix of the level of soluble factors in plasma and the percentage of **(a)** NK cells, **(b)** CD56dim NK cells, and **(c)** CD56bright NK cells in acute HFRS patients. Spearman correlation matrix of the clinical parameters and the percentage of **(d)** NK cells, **(e)** CD56dim NK cells, and **(f)** CD56bright NK cells in acute HFRS patients. The colour of the circles indicates positive (red) and negative (blue) correlations that were statistically significant (p < 0.05) as measured by the Spearman’s rank correlation coefficient test. The colour intensity and the size of the circle are proportional to the correlation coefficients. Ly: lymphocytes. Days a. symp.: days after symptoms onset.
(PDF)

S5 Fig ILCs display an activated and proliferating profile in peripheral blood of HFRS patients. **(a-g)** Representative flow cytometry plots and graphs showing the percentage of CD69+, Ki-67+, HLA-DR+, CD45RA+, CCR6+, CCR10+, and α4β7+ ILCs in control donors (n = 10) and HFRS patients during the acute (n = 15), early convalescence (n = 16), and late convalescence (n = 17) phase. **(h-j)** Spearman rank correlation between **(h)** plasma IL-10 levels and the percentage of CD69+ ILCs, **(i)** plasma CCL27 levels and the percentage of CCR10+ ILCs, and **(j)** plasma IL-7 levels and the percentage of Ki-67+ ILCs in acute HFRS patients (n = 15). **(k-m)** Spearman rank correlation between **(k)** plasma IL-10 levels and the percentage of CD69+ ILC2, **(l)** plasma CCL27 levels and the percentage of CCR10+ ILC2s, and **(m)** plasma TSLP levels and the percentage of CCR10+ ILC2s in acute HFRS patients (n = 15). Bar graphs are shown as mean and lines connect paired samples from the same patient. Statistical significance was assessed using the Wilcoxon signed-rank test to compare groups of HFRS patients, and the Kruskal-Wallis test followed by Dunn’s multiple comparisons test to compare controls with groups of HFRS patients. Severe patients are indicated by a black circle. ρ: Spearman´s rank correlation coefficient. *p < 0.05; **p < 0.01; ***p < 0.001; ****p < 0.0001.
(PDF)

S6 Fig Correlations of soluble factors and clinical parameters with ILCs in HFRS patients. Spearman correlation matrix of the level of soluble factors in plasma and the percentage of **(a)** ILCs, **(b)** ILC2s, and **(c)** nILCs in acute HFRS patients. Spearman correlation matrix of the clinical parameters and the percentage of **(d)** ILCs, **(e)** ILC2s, and **(f)** nILCs in acute HFRS patients. The colour of the circles indicates positive (red) and negative (blue) correlations that were statistically significant (p < 0.05) as measured by the Spearman’s rank correlation coefficient test. The colour intensity and the size of the circle are proportional to the correlation coefficients. Ly: lymphocytes. Days a. symp.: days after symptoms onset.
(PDF)

S7 Fig Type I IFNs are involved in ILC2 activation by PUUV-infected endothelial cells. Type I IFN blocking reagent B18R, anti-IL-6, anti-IL-12, anti-IL-18, and an IgG mouse isotype control for the blocking antibodies was added to supernatants of HUVEC cultures (PUUV-infected and uninfected) for 5 h prior to addition to human expanded ILC2s (n = 6, same donors as in Fig 6A–6D, 3 independent experiments) and incubation for 24 h. Bar graphs are shown as mean. Statistical significance was assessed using paired t-test. **p < 0.01.
(PDF)

S8 Fig ILC2s are a potential target of hantavirus infection *in vitro*. Relative fold change of viral RNA load in ILC2s exposed to PUUV calculated as a ratio of 2^ΔCt between 5 days post infection (dpi) and 5 hours post infection (hpi). Human expanded ILC2s (n = 3) were exposed to different multiplicities of infection (MOI) and samples collected at 5hpi and 5dpi and analyzed by RT-PCR. No change in viral load (a fold change of 1) is marked with a dotted line.
(PDF)

S1 Table Antibodies and reagents used for phenotyping. (PDF)

S2 Table Antibodies and reagents used for ILC2 phenotyping. (PDF)

S3 Table Antibodies and reagents used in flow cytometry for *in vitro* assays. (PDF)

S4 Table Extended clinical and laboratory characteristics of HFRS patients, acute phase. (PDF)

S1 Data Source data for figure graphs in this study. (XLSX)
