# Supplementary material for: Innate lymphoid cells are activated in HFRS, and their function can be modulated by hantavirus-induced type I interferons
Source: PLoS Pathog. 2024 Jul 22;20(7):e1012390. doi: 10.1371/journal.ppat.1012390 (PMC11293681; doi:10.1371/journal.ppat.1012390)
Supplement: S1 Fig — Gating strategy used for the identification of ILCs and NK cells by flow cytometry. (PDF) [file ppat.1012390.s001.pdf]

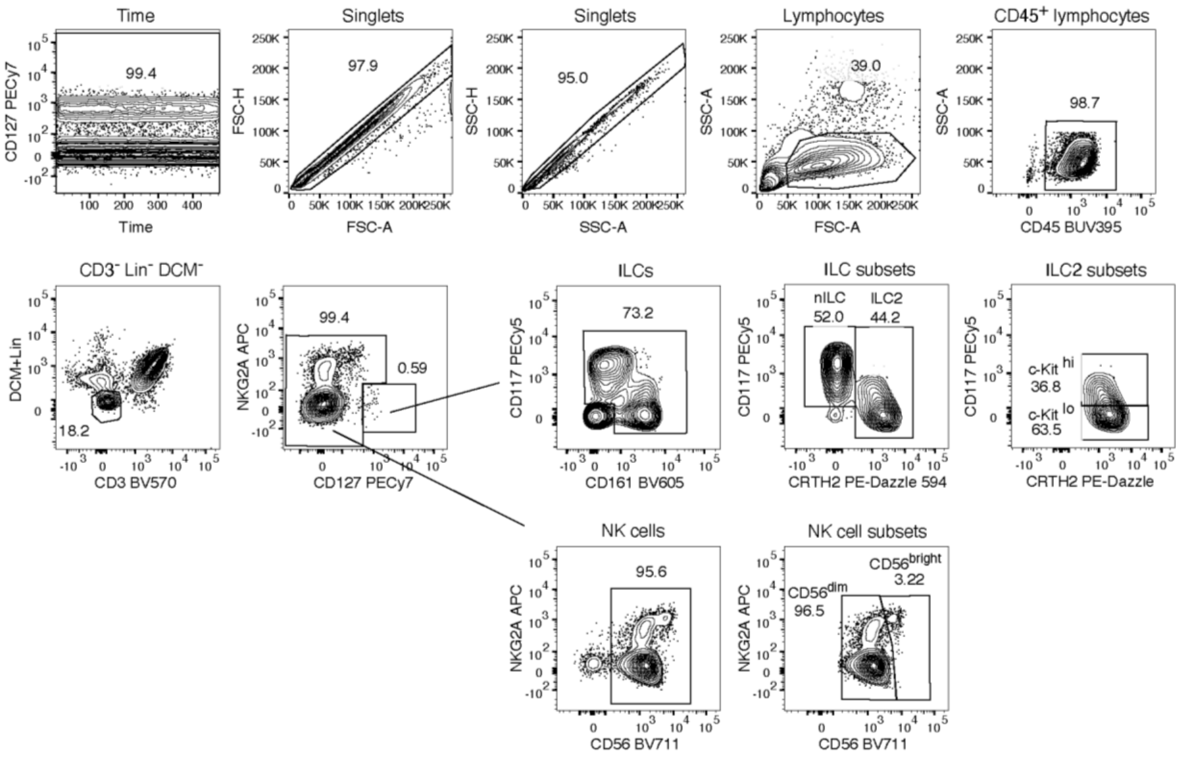

Supplementary Figure 1. Gating strategy for ILCs and NK cell identification in flow cytometry. Gating strategy used for the identification of ILCs and NK cells by flow cytometry.
