# Supplementary material for: Innate lymphoid cells are activated in HFRS, and their function can be modulated by hantavirus-induced type I interferons
Source: PLoS Pathog. 2024 Jul 22;20(7):e1012390. doi: 10.1371/journal.ppat.1012390 (PMC11293681; doi:10.1371/journal.ppat.1012390)
Supplement: S2 Fig — (a) Level of soluble factors in plasma of HFRS patients in acute (n = 15), early convalescence (n = 16), and late convalescence (n = 17) phase measured by multiplex immunoassay. Abbreviations: IL: interleukin. Bar graphs are shown as mean and lines connect paired samples from the same patient (circles). Statistical significance was assessed using the Wilcoxon signed-rank test. Severe patients are indicated by a black circle. (b) Spearman correlation matrix of the clinical parameters and the soluble markers measured in plasma of acute HFRS patients by a multiplex immunoassay. The colour of the circles indicates positive (red) and negative (blue) correlations that were statistically significant (p < 0.05) as measured by the Spearman’s rank correlation coefficient test. The colour intensity and the size of the circle are proportional to the correlation coefficients. (PDF) [file ppat.1012390.s002.pdf]

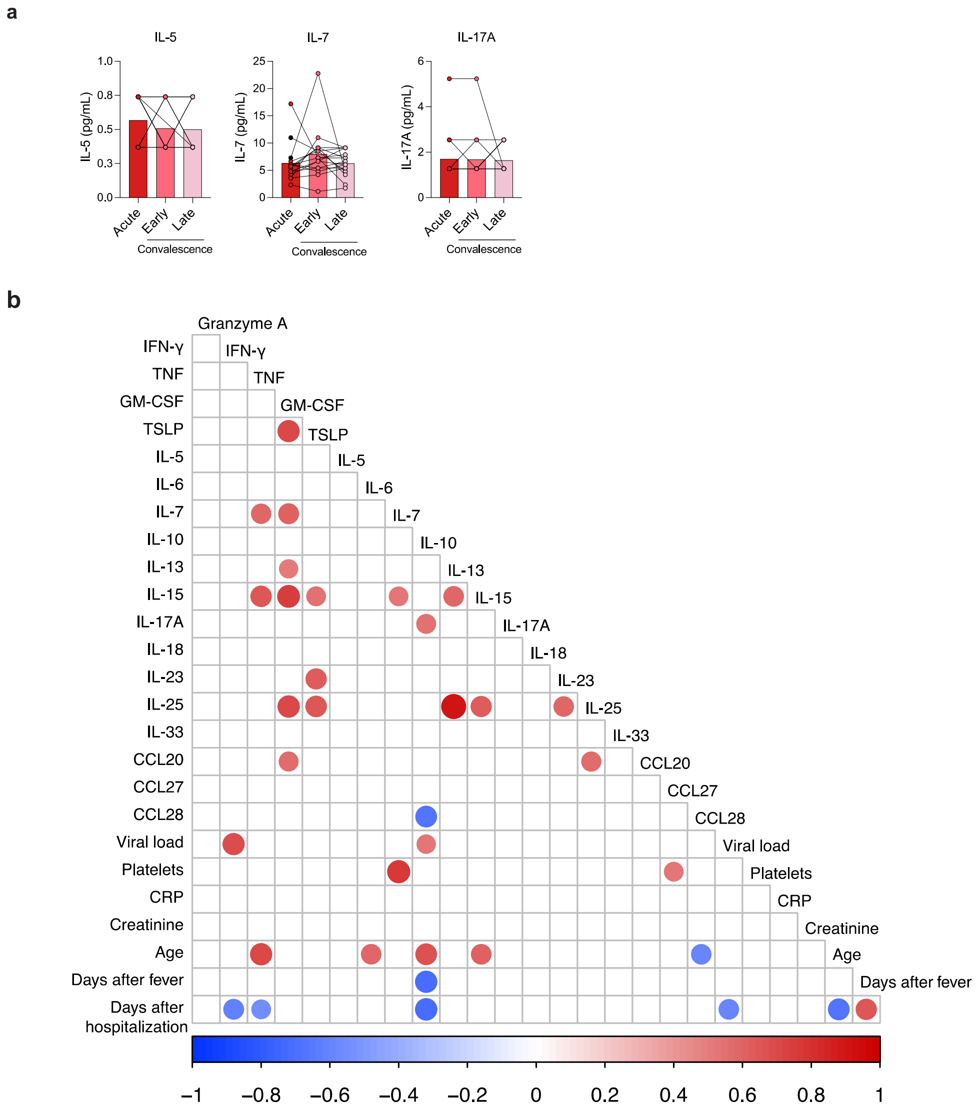

Supplementary Figure 2. Correlations of clinical parameters and soluble factors in plasma of HFRS patients.

(a) Level of soluble factors in plasma of HFRS patients in acute (n=15), early convalescence (n=16), and late convalescence (n=17) phase measured by multiplex immunoassay. Abbreviations: IL: interleukin. Bar graphs are shown as mean and lines connect paired samples from the same patient (circles). Statistical significance was assessed using the Wilcoxon signed-rank test. Severe patients are indicated by a black circle. **(b)** Spearman correlation matrix of the clinical parameters and the soluble markers measured in plasma of acute HFRS patients by a multiplex immunoassay. The colour of the circles indicates positive (red) and negative (blue) correlations that were statistically significant ($p < 0.05$) as measured by the Spearman's rank correlation coefficient test. The colour intensity and the size of the circle are proportional to the correlation coefficients.
