# Supplementary material for: Innate lymphoid cells are activated in HFRS, and their function can be modulated by hantavirus-induced type I interferons
Source: PLoS Pathog. 2024 Jul 22;20(7):e1012390. doi: 10.1371/journal.ppat.1012390 (PMC11293681; doi:10.1371/journal.ppat.1012390)
Supplement: S3 Fig — (a-b) Percentage of CD69+, Ki-67+, HLA-DR+, NKp44+, NKG2A+, CCR6+, CCR10+, α4β7+, CD45RA+, and CD161+ (a) CD56dim NK cells and (b) CD56bright NK cells in control donors (n = 10) and HFRS patients during the acute (n = 15), early convalescence (n = 16), and late convalescence (n = 17) phase. (c-e) Spearman rank correlation between (c) plasma IL-10 levels and the percentage of Ki-67+ NK cells, (d) plasma granzyme A (GrzA) levels and the percentage of CD69+ NK cells, and (e) plasma viral load (n = 13; PUUV S RNA copies/mL) and the percentage of NK cells out of CD45+ lymphocytes in acute HFRS patients. Bar graphs are shown as mean and lines connect paired samples from the same patient. Statistical significance was assessed using the Wilcoxon signed-rank test to compare groups of HFRS patients, and the Kruskal-Wallis test followed by Dunn’s multiple comparisons test to compare controls with groups of HFRS patients. Severe patients are indicated by a black circle. ρ: Spearman´s rank correlation coefficient. *p < 0.05; **p < 0.01; ***p < 0.001; ****p < 0.0001. (PDF) [file ppat.1012390.s003.pdf]

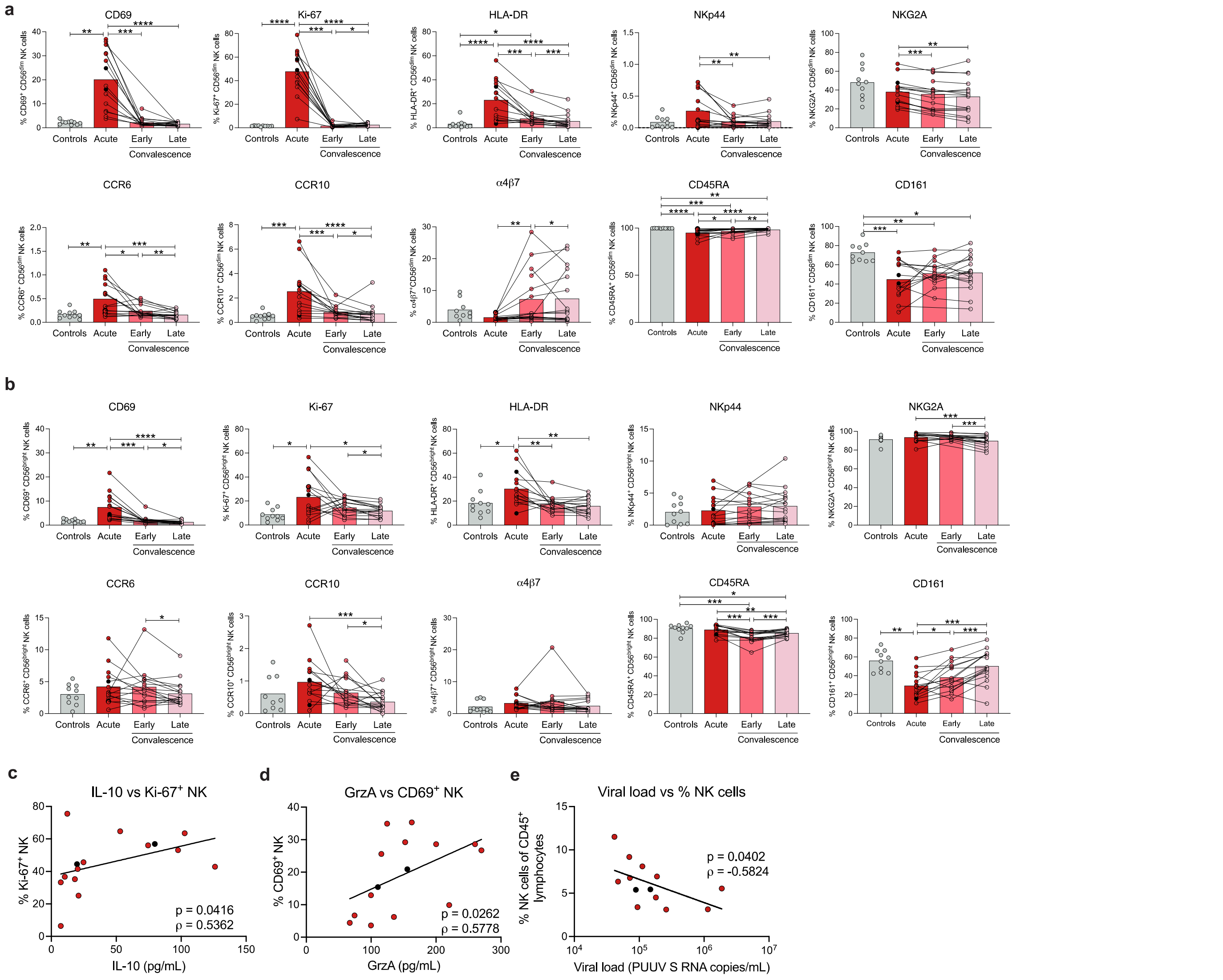

Supplementary Figure 3. NK cell subsets are activated, proliferating, and present a migratory profile in peripheral blood of HFRS patients.

(a-b) Percentage of $CD69^{+}$, Ki-67^{+}, HLA-DR^{+}, $NKp44^{+}$, $NKG2A^{+}$, $CCR6^{+}$, $CCR10^{+}$, $\alpha4\beta7^{+}$, $CD45RA^{+}$, and $CD161^{+}$ **(a)** $CD56^{dim}$ NK cells and **(b)** $CD56^{bright}$ NK cells in control donors (n=10) and HFRS patients during the acute (n=15), early convalescence (n=16), and late convalescence (n=17) phase.

(c-e) Spearman rank correlation between **(c)** plasma IL-10 levels and the percentage of Ki-67^{+} NK cells, **(d)** plasma granzyme A (GrzA) levels and the percentage of $CD69^{+}$ NK cells, and **(e)** plasma viral load (n=13; PUUV S RNA copies/mL) and the percentage of NK cells out of $CD45^{+}$ lymphocytes in acute HFRS patients. Bar graphs are shown as mean and lines connect paired samples from the same patient. Statistical significance was assessed using the Wilcoxon signed-rank test to compare groups of HFRS patients, and the Kruskal-Wallis test followed by Dunn's multiple comparisons test to compare controls with groups of HFRS patients. Severe patients are indicated by a black circle. ρ: Spearman´s rank correlation coefficient. *p < 0.05; **p < 0.01; ***p < 0.001; ****p < 0.0001.
