# Supplementary material for: Innate lymphoid cells are activated in HFRS, and their function can be modulated by hantavirus-induced type I interferons
Source: PLoS Pathog. 2024 Jul 22;20(7):e1012390. doi: 10.1371/journal.ppat.1012390 (PMC11293681; doi:10.1371/journal.ppat.1012390)
Supplement: S5 Fig — (a-g) Representative flow cytometry plots and graphs showing the percentage of CD69+, Ki-67+, HLA-DR+, CD45RA+, CCR6+, CCR10+, and α4β7+ ILCs in control donors (n = 10) and HFRS patients during the acute (n = 15), early convalescence (n = 16), and late convalescence (n = 17) phase. (h-j) Spearman rank correlation between (h) plasma IL-10 levels and the percentage of CD69+ ILCs, (i) plasma CCL27 levels and the percentage of CCR10+ ILCs, and (j) plasma IL-7 levels and the percentage of Ki-67+ ILCs in acute HFRS patients (n = 15). (k-m) Spearman rank correlation between (k) plasma IL-10 levels and the percentage of CD69+ ILC2, (l) plasma CCL27 levels and the percentage of CCR10+ ILC2s, and (m) plasma TSLP levels and the percentage of CCR10+ ILC2s in acute HFRS patients (n = 15). Bar graphs are shown as mean and lines connect paired samples from the same patient. Statistical significance was assessed using the Wilcoxon signed-rank test to compare groups of HFRS patients, and the Kruskal-Wallis test followed by Dunn’s multiple comparisons test to compare controls with groups of HFRS patients. Severe patients are indicated by a black circle. ρ: Spearman´s rank correlation coefficient. *p < 0.05; **p < 0.01; ***p < 0.001; ****p < 0.0001. (PDF) [file ppat.1012390.s005.pdf]

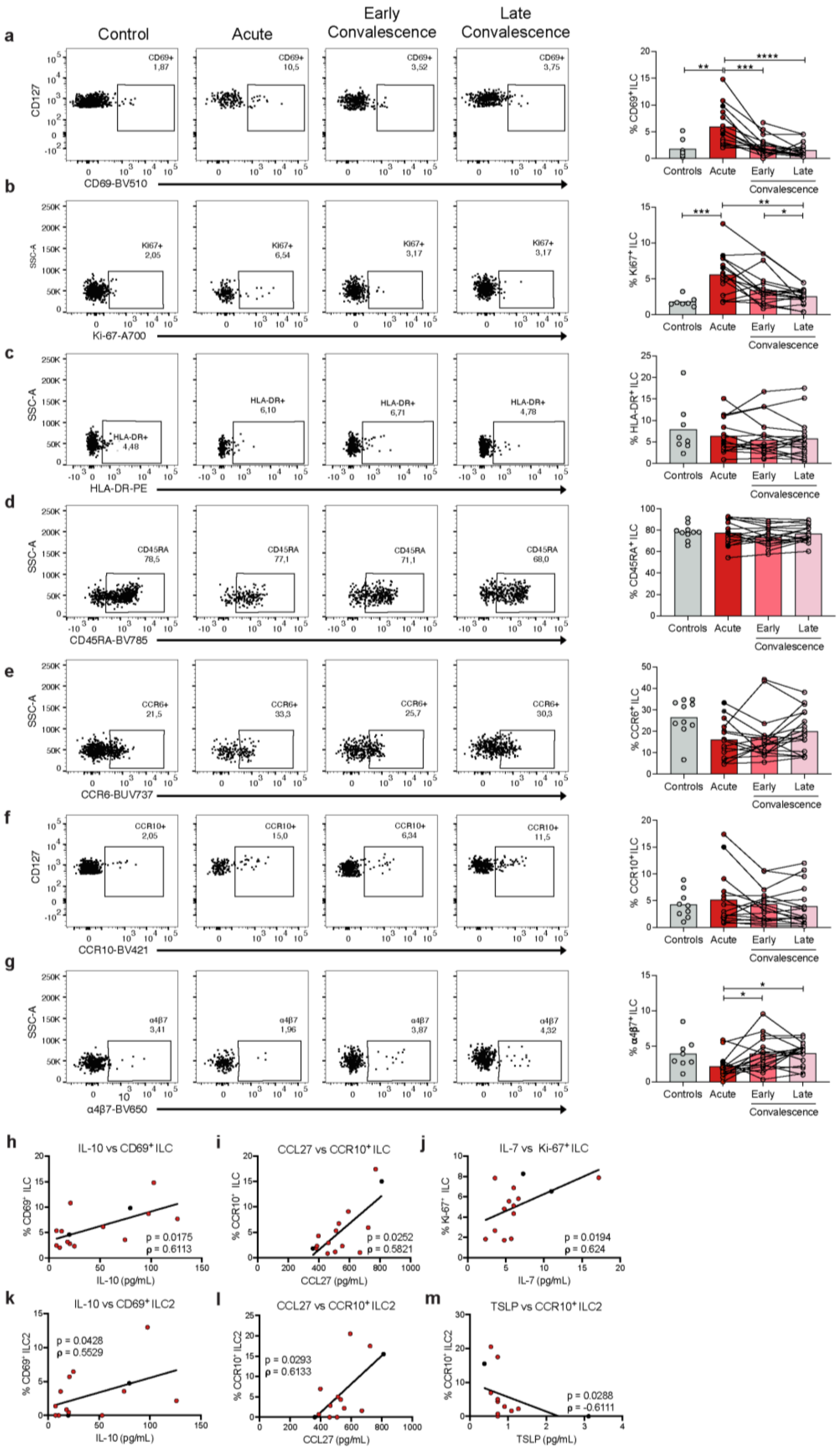

Supplementary Figure 5. ILCs display an activated and proliferating profile in peripheral blood of HFRS patients.

(a-g) Representative flow cytometry plots and graphs showing the percentage of CD69^{+}, Ki-67^{+}, HLA-DR^{+}, CD45RA^{+}, CCR6^{+}, CCR10^{+}, and α4β7^{+} ILCs in control donors (n=10) and HFRS patients during the acute (n=15), early convalescence (n=16), and late convalescence (n=17) phase.

(h-j) Spearman rank correlation between **(h)** plasma IL-10 levels and the percentage of CD69^{+} ILCs, **(i)** plasma CCL27 levels and the percentage of CCR10^{+} ILCs, and **(j)** plasma IL-7 levels and the percentage of Ki-67^{+} ILCs in acute HFRS patients (n = 15).

(k-m) Spearman rank correlation between **(k)** plasma IL-10 levels and the percentage of $CD69^+$ ILC2, **(l)** plasma CCL27 levels and the percentage of $CCR10^+$ ILC2s, and **(m)** plasma TSLP levels and the percentage of $CCR10^+$ ILC2s in acute HFRS patients (n = 15).

Bar graphs are shown as mean and lines connect paired samples from the same patient. Statistical significance was assessed using the Wilcoxon signed-rank test to compare groups of HFRS patients, and the Kruskal-Wallis test followed by Dunn's multiple comparisons test to compare controls with groups of HFRS patients. Severe patients are indicated by a black circle. ρ: Spearman´s rank correlation coefficient. $*p < 0.05$; $**p < 0.01$; $***p < 0.001$; $****p < 0.0001$.
