# Supplementary material for: Innate lymphoid cells are activated in HFRS, and their function can be modulated by hantavirus-induced type I interferons
Source: PLoS Pathog. 2024 Jul 22;20(7):e1012390. doi: 10.1371/journal.ppat.1012390 (PMC11293681; doi:10.1371/journal.ppat.1012390)
Supplement: S6 Fig — Spearman correlation matrix of the level of soluble factors in plasma and the percentage of (a) ILCs, (b) ILC2s, and (c) nILCs in acute HFRS patients. Spearman correlation matrix of the clinical parameters and the percentage of (d) ILCs, (e) ILC2s, and (f) nILCs in acute HFRS patients. The colour of the circles indicates positive (red) and negative (blue) correlations that were statistically significant (p < 0.05) as measured by the Spearman’s rank correlation coefficient test. The colour intensity and the size of the circle are proportional to the correlation coefficients. Ly: lymphocytes. Days a. symp.: days after symptoms onset. (PDF) [file ppat.1012390.s006.pdf]

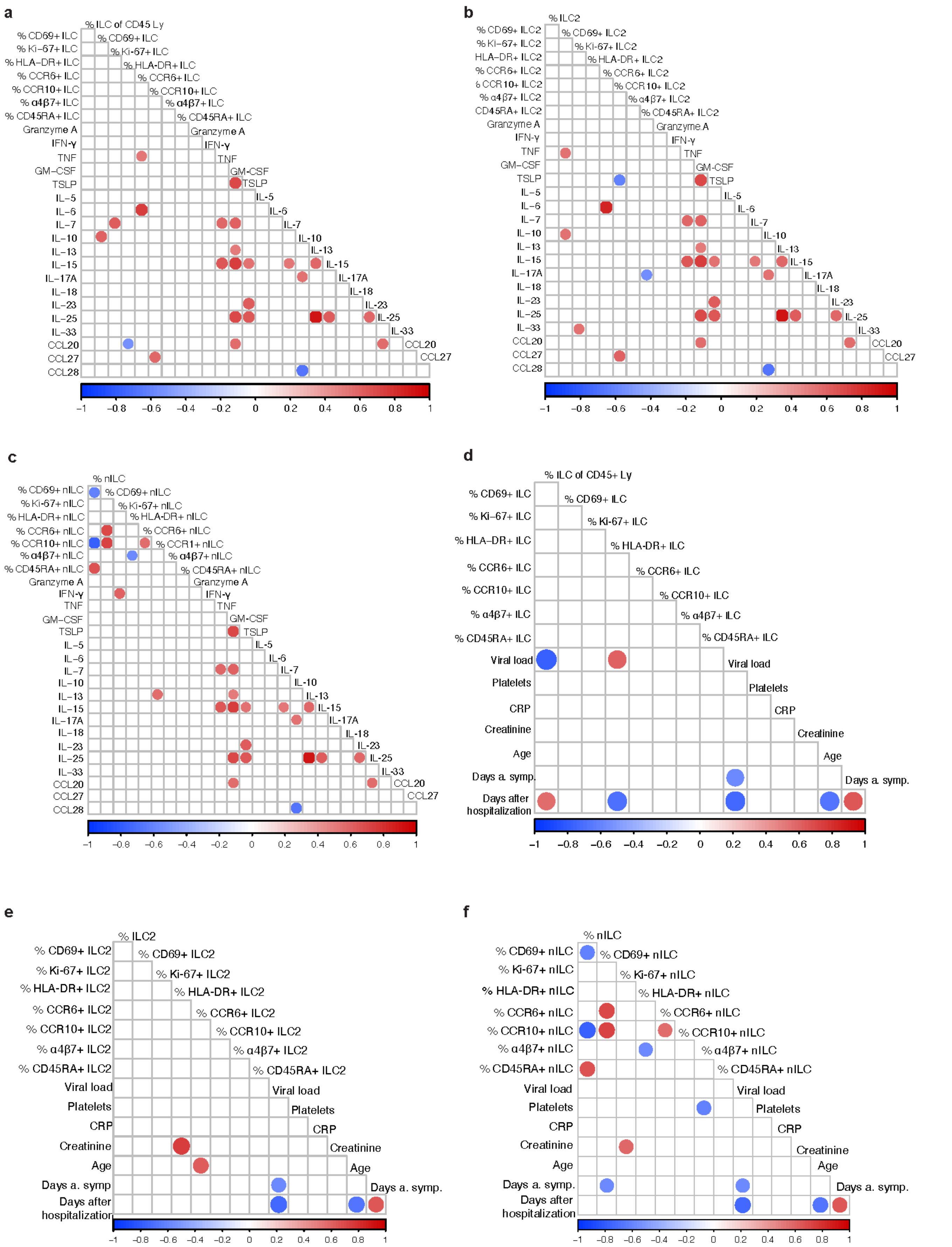

Supplementary Figure 6. Correlations of soluble factors and clinical parameters with ILCs in HFRS patients.

Spearman correlation matrix of the level of soluble factors in plasma and the percentage of **(a)** ILCs, **(b)** ILC2s, and **(c)** nILCs in acute HFRS patients. Spearman correlation matrix of the clinical parameters and the percentage of **(d)** ILCs, **(e)** ILC2s, and **(f)** nILCs in acute HFRS patients. The colour of the circles indicates positive (red) and negative (blue) correlations that were statistically significant ($p < 0.05$) as measured by the

Spearman’s rank correlation coefficient test. The colour intensity and the size of the circle are proportional to the correlation coefficients. Ly: lymphocytes. Days a. symp.: days after symptoms onset.
