# Supplementary material for: Innate lymphoid cells are activated in HFRS, and their function can be modulated by hantavirus-induced type I interferons
Source: PLoS Pathog. 2024 Jul 22;20(7):e1012390. doi: 10.1371/journal.ppat.1012390 (PMC11293681; doi:10.1371/journal.ppat.1012390)
Supplement: S7 Fig — Type I IFN blocking reagent B18R, anti-IL-6, anti-IL-12, anti-IL-18, and an IgG mouse isotype control for the blocking antibodies was added to supernatants of HUVEC cultures (PUUV-infected and uninfected) for 5 h prior to addition to human expanded ILC2s (n = 6, same donors as in Fig 6A–6D, 3 independent experiments) and incubation for 24 h. Bar graphs are shown as mean. Statistical significance was assessed using paired t-test. **p < 0.01. (PDF) [file ppat.1012390.s007.pdf]

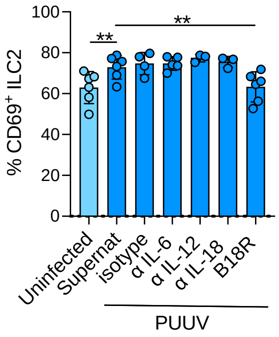

Supplementary Figure 7. Type I IFNs are involved in ILC2 activation by PUUV-infected endothelial cells.

Type I IFN blocking reagent B18R, anti-IL-6, anti-IL-12, anti-IL-18, and an IgG mouse isotype control for the blocking antibodies was added to supernatants of HUVEC cultures (PUUV-infected and uninfected) for 5 h prior to addition to human expanded ILC2s (n = 6, same donors as in Fig 6a-d, 3 independent experiments) and incubation for 24 h. Bar graphs are shown as mean. Statistical significance was assessed using paired t-test. **p < 0.01.
