# Supplementary material for: Innate lymphoid cells are activated in HFRS, and their function can be modulated by hantavirus-induced type I interferons
Source: PLoS Pathog. 2024 Jul 22;20(7):e1012390. doi: 10.1371/journal.ppat.1012390 (PMC11293681; doi:10.1371/journal.ppat.1012390)
Supplement: S8 Fig — Relative fold change of viral RNA load in ILC2s exposed to PUUV calculated as a ratio of 2^ΔCt between 5 days post infection (dpi) and 5 hours post infection (hpi). Human expanded ILC2s (n = 3) were exposed to different multiplicities of infection (MOI) and samples collected at 5hpi and 5dpi and analyzed by RT-PCR. No change in viral load (a fold change of 1) is marked with a dotted line. (PDF) [file ppat.1012390.s008.pdf]

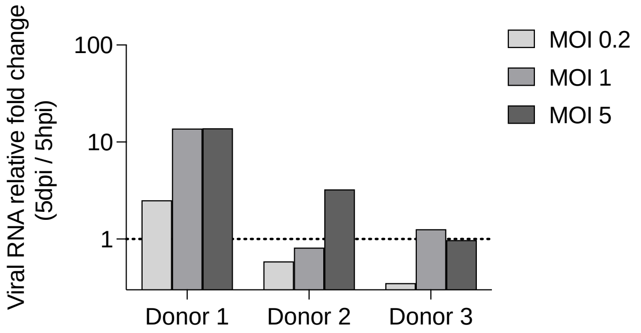

Supplementary Figure 8. ILC2s are a potential target of hantavirus infection *in vitro*.
Relative fold change of viral RNA load in ILC2s exposed to PUUV calculated as a ratio of $2^{\wedge\Delta Ct}$ between 5 days post infection (dpi) and 5 hours post infection (hpi). Human expanded ILC2s (n=3) were exposed to different multiplicities of infection (MOI) and samples collected at 5hpi and 5dpi and analyzed by RT-PCR. No change in viral load (a fold change of 1) is marked with a dotted line.
