# Supplementary material for: Innate lymphoid cells are activated in HFRS, and their function can be modulated by hantavirus-induced type I interferons
Source: PLoS Pathog. 2024 Jul 22;20(7):e1012390. doi: 10.1371/journal.ppat.1012390 (PMC11293681; doi:10.1371/journal.ppat.1012390)
Supplement: S1 Table — (PDF) [file ppat.1012390.s009.pdf]

Supplementary Table 1. Antibodies and reagents used for phenotyping

Fluorochrome	Marker	Clone	Company	Catalog number	RRID
BUV395	CD45	HI30	BD	563791	AB_2744400
BUV737	CCR6	11A9	BD	612780	AB_2870109
FITC	CD1a	HI149	Biolegend	300104	AB_314018
	CD14	Tuk4	Life Technologies	MHCD14014	AB_1464899
	CD19	4G7	BD Biosciences	345776	AB_2868804
	CD34	581	Biolegend	343504	AB_1731852
	CD123	6H6	Biolegend	306014	AB_2124259
	CD303 (BDCA2)	AC144	Miltenyi	130-113-192	AB_2726017
	FcεR1α	AER-37 (CRA1)	Biolegend	334608	AB_1227653
	TCRαβ	IP26	Biolegend	306706	AB_314644
	TCRgγδ	B1	Biolegend	331208	AB_1575108
	Dead Cell Marker		ThermoFischer Scientific	L23101	N/A
APC	NKG2A	Z199	Beckman Coulter	A60797	AB_10643105
A700	Ki-67	B56	BD	561277	AB_10611571
BV421	CCR10	1B5	BD	564770	AB_2738942
BV510	CD69	FN50	Biolegend	747521	N/A
BV570	CD3	UCHT1	Biolegend	300436	AB_2562124
BV605	CD161	HP-3810	Biolegend	339916	AB_2563607
BV650	α4β7 biotin	HU117	R&D	MAB10078-100	N/A
	streptavidin		Biolegend	405231	N/A
BV711	CD56	HCD56	Biolegend	318336	AB_2562417
BV785	CD45RA	HI100	Biolegend	304140	AB_2563816
PE	HLADR	L243	Biolegend	307606	AB_314684
PE-Dazzle 594	CRTH2	BM16	Biolegend	350125	AB_2572052
PE-Cy5	NKp44	Z231	Beckman Coulter	A66903	N/A
PE-Cy5.5	CD117	104D2D1	Beckman Coulter	B96754	N/A
PE-Cy7	CD127	R34.34	Beckman Coulter	A64618	AB_2833031
