# Supplementary material for: Innate lymphoid cells are activated in HFRS, and their function can be modulated by hantavirus-induced type I interferons
Source: PLoS Pathog. 2024 Jul 22;20(7):e1012390. doi: 10.1371/journal.ppat.1012390 (PMC11293681; doi:10.1371/journal.ppat.1012390)
Supplement: S2 Table — (PDF) [file ppat.1012390.s010.pdf]

Supplementary Table 2. Antibodies and reagents used for ILC2s phenotyping.

**Fluorochrome**	**Marker**	**Clone**	**Company**	**Catalog number**	**RRID**
FITC	CD1a	HI149	Biolegend	300104	AB_314018
	CD14	Tuk4	Life Technologies	MHCD14014	AB_1464899
	CD19	4G7	BD Biosciences	345776	AB_2868804
	CD34	581	Biolegend	343504	AB_1731852
	CD94	DX22	Biolegend	305504	AB_314534
	CD123	6H6	Biolegend	306014	AB_2124259
	CD303 (BDCA2)	AC144	Miltenyi	130-113-192	AB_2726017
	FcεR1α	AER-37 (CRA1)	Biolegend	334608	AB_1227653
	TCRαβ	IP26	Biolegend	306706	AB_314644
	TCRγδ	B1	Biolegend	331208	AB_1575108
	Dead Cell Marker		ThermoFischer Scientific	L23101	N/A
PE-Dazzle 594	CRTH2	BM16	Biolegend	350125	AB_2572052
PE-Cy5.5	CD117	104D2D1	Beckman Coulter	B96754	N/A
PE-Cy7	CD127	R34.34	Beckman Coulter	A64618	AB_2833031
BV510	CD45 V500	HI30	BD Biosciences	560777	AB_1937324
BV605	CD161	HP-3810	Biolegend	339916	AB_2563607
BV785	CD3	OKT3	Biolegend	317330	AB_2563507

N/A: not available
