# Supplementary material for: Innate lymphoid cells are activated in HFRS, and their function can be modulated by hantavirus-induced type I interferons
Source: PLoS Pathog. 2024 Jul 22;20(7):e1012390. doi: 10.1371/journal.ppat.1012390 (PMC11293681; doi:10.1371/journal.ppat.1012390)
Supplement: S3 Table — (PDF) [file ppat.1012390.s011.pdf]

Supplementary Table 3. Antibodies and reagents used in flow cytometry for *in vitro* assays.

Fluorochrome	Marker	Clone	Company	Catalog number	RRID
BUV395	CD45	HI30	BD	563791	AB_2744400
BUV737	CCR6	11A9	BD	612780	AB_2870109
FITC	DCM		ThermoFischer Scientific	L23101	N/A
BV510	CD69	FN50	Biolegend	747521	N/A
BV570	CD3	UCHT1	Biolegend	300436	AB_2562124
BV785	CD45RA	HI100	Biolegend	304140	AB_2563816
PE-Cy5.5	CD117	104D2D1	Beckman Coulter	B96754	N/A

N/A: not available
