# Supplementary material for: Innate lymphoid cells are activated in HFRS, and their function can be modulated by hantavirus-induced type I interferons
Source: PLoS Pathog. 2024 Jul 22;20(7):e1012390. doi: 10.1371/journal.ppat.1012390 (PMC11293681; doi:10.1371/journal.ppat.1012390)
Supplement: S4 Table — (PDF) [file ppat.1012390.s012.pdf]

Supplementary Table 4. Extended clinical and laboratory characteristics of HFRS patients, acute phase

Age, years	Sex	Days after symptoms onset	Days after hosp.	Days hosp.	PUUV*	Leucocyte count (10^9/L)	Hematocrit (L/L)	Platelet count (10^9/L)	CRP (mg/L)	Creatinine (µmol/L)	CMV**	EBV**	MAP	Severity score	Severity
58	F	7	1	6	187975	9	0.35	114	81.7	54	neg	neg	82	1	Mild
48	M	6	2	9	88955	21.8	0.44	91	87.3	519	neg	338	92	6	Sev ere
30	F	9	4	5	71901	8.8	0.36	131	43.1	191	neg	neg	74	3	Mild
38	M	6	1	2	1867667	10.3	0.44	52	111.2	120	neg	neg	92	3	Mild
54	M	6	1	4	94272	12.2	0.46	89	27	179	neg	neg	83	4	Mild
41	M	6	1	4	149025	8.2	0.36	121	94	498	neg	neg	82	5	Severe
30	F	8	3	5	70523	7.5	0.38	102	20	85	neg	neg	72	1	Mild
25	F	8	3	NA	112205	18.2	0.36	86	58.2	107	neg	neg	68	3	Mild
67	F	6	1	9	1143969	10.8	0.34	118	33.5	431	neg	neg	76	4	Mild
55	F	6	2	7	ND	12	0.37	82	33.4	254	neg	neg	98	4	Mild
29	F	7	3	6	47300	6.9	0.35	66	94	155	neg	neg	91	3	Mild
28	F	8	2	4	ND	5.7	0.34	199	24.7	137	neg	neg	100	1	Mild
35	F	5	2	5	181000	NA	NA	NA	93.5	42	neg	neg	83	0	Mild
28	F	6	2	4	261000	8.6	0.4	115	51.8	NA	neg	neg	76	1	Mild
35	M	7	3	NA	41600	9.5	0.37	86	78.5	99	neg	neg	90	2	Mild

F, female; M, male; hosp., hospitalization; NA, not available; ND, not detectable; neg, negative; CMV, Cytomegalovirus; EBV, Epstein-Barr virus; MAP, mean arterial blood pressure.
*PUUV S RNA copies/mL
** copies/mL.
Platelet count; normal range 150–360 x10^9/L.
Plasma C-reactive protein (CRP); reference <3 mg/L.
Plasma creatinine; reference <90 µmol/L for women, <100 µmol/L for men.
Mean arterial blood pressure (MAP); reference 70–100 mmHg.
